# Genetic architecture of cyst nematode resistance revealed by genome-wide association study in soybean

**DOI:** 10.1186/s12864-015-1811-y

**Published:** 2015-08-12

**Authors:** T. D. Vuong, H. Sonah, C. G. Meinhardt, R. Deshmukh, S. Kadam, R. L. Nelson, J. G. Shannon, H. T. Nguyen

**Affiliations:** Division of Plant Sciences and National Center for Soybean Biotechnology (NCSB), University of Missouri, Columbia, MO 65211 USA; Soybean Maize Germplasm, Pathology, and Genetics Research Unit, USDA, Agricultural Research Service, and Department of Crop Sciences University of Illinois, Urbana, IL 61801 USA; Division of Plant Sciences and NCSB, University of Missouri, Portageville, MO 63873 USA; Present address: Département de Phytologie, Faculté des Sciences de l’Agriculture et de l’Alimentation, Centre de Recherche en Horticulture, Université Laval, Quebec, Canada

**Keywords:** Soybean *(Glycine max)*, Genome-wide association study (GWAS), Soybean cyst nematode (SCN), Linkage disequilibrium (LD), Single nucleotide polymorphisms (SNP)

## Abstract

**Background:**

Bi-parental mapping populations have been commonly utilized to identify and characterize quantitative trait loci (QTL) controlling resistance to soybean cyst nematode (SCN, *Heterodera glycines* Ichinohe). Although this approach successfully mapped a large number of SCN resistance QTL, it captures only limited allelic diversity that exists in parental lines, and it also has limitations for genomic resolution. In this study, a genome-wide association study (GWAS) was performed using a diverse set of 553 soybean plant introductions (PIs) belonging to maturity groups from III to V to detect QTL/genes associated with SCN resistance to HG Type 0.

**Results:**

Over 45,000 single nucleotide polymorphism (SNP) markers generated by the SoySNP50K iSelect BeadChip (*http//**www.soybase.org*) were utilized for analysis. GWAS identified 14 loci distributed over different chromosomes comprising 60 SNPs significantly associated with SCN resistance. Results also confirmed six QTL that were previously mapped using bi-parental populations, including the *rhg1* and *Rhg4* loci. GWAS identified eight novel QTL, including QTL on chromosome 10, which we have previously mapped by using a bi-parental population. In addition to the known loci for four simple traits, such as seed coat color, flower color, pubescence color, and stem growth habit, two traits, like lodging and pod shattering, having moderately complex inheritance have been confirmed with great precision by GWAS.

**Conclusions:**

The study showed that GWAS can be employed as an effective strategy for identifying complex traits in soybean and for narrowing GWAS-defined genomic regions, which facilitates positional cloning of the causal gene(s).

**Electronic supplementary material:**

The online version of this article (doi:10.1186/s12864-015-1811-y) contains supplementary material, which is available to authorized users.

## Background

Soybean cyst nematode (SCN, *Heterodera glycines* Ichinohe) is one of the most devastating plant-parasitic nematode species causing severe annual soybean yield losses worldwide. It was estimated that this nematode species causes nearly $1 billion annually in yield losses in the United States soybean production alone [1]. Once established in a soybean field, it is very difficult to eradicate an SCN infestation because of the fact that among potential reasons the genetic diversity of *H. glycines* field populations and their ability to eventually overcome resistance genes of host plants. The identification and utilization of new sources of resistance to develop SCN-resistant varieties have been demonstrated to be most efficient and practical manner to control this nematode. However, most SCN-resistant varieties have been derived from a few common resistance sources, namely plant introductions (PIs) 88788 and 548402 (Peking). Diers and Arelli [1] reported over 80 % of public cultivars released during the 1990s with SCN-resistance were derived from PI 88788 alone in the north-central US. A similar trend was also observed for SCN-resistant cultivars developed by private industry. Thus, it has been shown that the continuous cultivation of the same source of resistance has resulted in genetic shifts of SCN populations. Mitchum et al. [2] reported results of a survey showing that most of the SCN populations collected from Missouri soybean fields were virulent or could reproduce on indicator lines, like PI 88788, PI 209332, PI 548316, and Peking, used as resistance sources for soybean cultivars. Lack of diversity for SCN resistance genes in soybean cultivars requires further investigation to identify new SCN genes from other sources of resistance [3].

Early studies of the inheritance of resistance to SCN indicated that SCN resistance was genetically controlled by different recessive or dominant genes, designated as *rhg1*, *rhg2*, *rhg3* [4], *Rhg4* [5], and *Rhg5* [6]. However, further genetic studies of new resistance sources have showed that SCN resistance was a complex trait genetically controlled by quantitative trait loci (QTL) [7, 8]. In a comprehensive review, Concibido et al. [9] has summarized 31 putative QTL associated with resistance to various SCN HG types, which were mapped to 17 of the 20 soybean chromosomes. With new resistance sources, many efforts have been made to identify novel QTL, which were mapped on new loci [3, 10]. In addition to the identification of new QTL, genetic analysis also confirmed many QTL previously reported [11, 12]. Recently, two major genomic loci, *rhg1* and *Rhg4*, which have been identified and consistently mapped on chromosomes (Chrs.) 18 and 8, respectively, were successfully cloned [13, 14].

For decades, QTL mapping, also known as linkage mapping, has been well-established and demonstrated to be a powerful tool for studying the genetic basis of complex quantitative traits in plants. Mapping populations derived from bi-parental crosses have been commonly utilized to identify and genetically map causative genomic locations for various biotic and abiotic stress related traits, leading to successful map-based cloning and candidate gene identification [15–19]. However, this method has limitations because it only captures limited allelic diversity existing in two parental lines. It is also limited in genomic resolution provided by low recombination events incurring during population development. Recently, genome-wide association studies (GWAS), which have been long carried out in human genetic research with the great advantages over the linkage mapping, have been adapted and proved to be an alternative mapping approach in identifying and dissecting significant QTL regions harboring candidate genes of interest in plants [20]. Plant GWAS is gaining popularity because of advances in genome sequencing technologies. Moreover, compared with linkage mapping, plant GWAS enables the investigation of a set of genetically unstructured genotypes, and if a sufficient number of genetic markers are used, it can generate more precise QTL positions. A large number of GWAS has been successfully conducted in many plant species, such as *Arabidopsis* [21], rice [22], maize [23], barley [24], tomato [25], oat [26], and sorghum [27].

In soybean, efforts have been made using GWAS to detect and characterize QTL conveying a number of traits of interest for the past several years. Wang et al. [28] studied iron deficiency chlorosis (IDC) using simple single repeat (SSR) markers in two advanced breeding line populations. The authors identified and confirmed several markers significantly associated with IDC. Also using SSR markers in a study of seed protein content, Jun et al. [29] not only detected previously reported QTL and associated genetic markers, but also identified new genomic regions that were not reported in earlier genetic analysis. These GWAS efforts, conducted with few markers, have limitations for mapping resolution and genome coverage. With the successful adaptation of the genotyping-by-sequencing (GBS) method in soybean, Sonah et al. and Bastien et al. [30, 31] has performed a GWAS in a collection of 130 soybean breeding lines for resistance to Sclerotinia stem rot (*Sclerotinia sclerotiorum*). The authors identified very significantly associated locus on chromosome (Chr.) 15 governing resistance to Sclerotinia stem rot, and subsequently performed candidate genes identification in this region. More recently, Hwang et al. [32] conducted a GWAS for seed protein and oil content using over 55,000 SNPs in a diverse set of 298 soybean accessions. The study not only identified most of the previously reported QTL for seed protein and oil content, but also greatly narrowed down these genomic regions. Of these, the well-known major QTL region on Chr. 20 for high protein content was detected. Sonah et al. [33] performed a GWAS for oil and protein content along with the six morphological simple traits using over 17,000 SNPs developed by GBS approaches in a subset of 139 short-season soybean lines. With such high resolution marker coverage, the authors successfully identified a highly significant association for the SNPs in the candidate genes. In some cases, the identified QTL were subsequently validated in further genetic analysis using traditional bi-parental mapping populations.

With respect to SCN, GWAS has also been performed to identify genomic regions for resistance. Among association mapping work in soybean, Li et al. [34] studied a set of 159 soybean accessions genotyped with 55 SSR markers. The authors identified and located six SSRs significantly associated with SCN resistance on different chromosomes. More recently, Bao et al. [35] conducted association mapping of 282 soybean breeding lines representative of the University of Minnesota soybean breeding program for resistance to SCN HG Type 0, using the USLP 1.0 SNP arrays [36]. The association mapping detected significant association of the known genes, *rhg1* and *FGAM1,* and a third locus located at the opposite end of Chr. 18. The authors concluded that association mapping can be an effective genomic tool for identifying genes of interest in diverse germplasm.

In an effort to discover new sources of broad-based resistance to SCN, a diverse panel of 553 soybean germplasm accessions, which were undergone multivariate selection procedures and best represents the diversity of the total collection, was evaluated for response to SCN HG Type 0 [37]. Preliminary analysis identified over 40 new germplasm accessions with moderate to high resistance to different SCN HG Types (Nguyen Lab, unpublished data); however, no genetic analysis had been conducted to detect and map QTL/genes controlling SCN resistance in these accessions. The objectives of the present study were (i) to conduct a GWAS to detect novel QTL and to confirm the known QTL associated with resistance to SCN in the diverse panel of soybean germplasm accessions and, (ii) to identify candidate genes harbored in the causative genomic locations aiming to enhancing understanding molecular mechanism of SCN resistance and facilitating map-based cloning of the genes identified.

## Results

### Frequency distribution and source of resistance to SCN HG Type 0

**Fig. 1:**
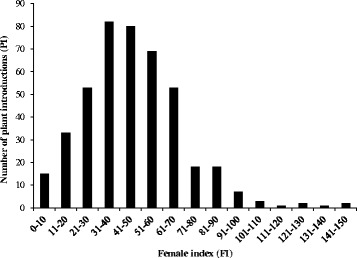
Frequency distribution of female index (FI) in a diverse panel of 553 soybean germplasm accessions evaluated in this study

Greenhouse evaluations of a diverse set of germplasm for resistance to SCN HG Type 0 revealed a very broad range of female index (FI) scoring (from 0 to 145) and showed a normal distribution (Fig. 1). Only 24 genotypes showed a high level of resistance (FI <10) against HG Type 0. Comparatively, very few (18) genotypes showed moderate resistance ranging from 10 to 30. Further characterization of high and moderate resistant genotypes categorized Peking-type and PI 88788-type resistant sources. The germplasm set evaluated in this study included 26 PI lines known for SCN resistance. In addition, the study has identified 10 new PIs showing a high level of resistance (Additional file 1: Table S1).

### Phylogeny, population structure and kinship among the SCN resistant soybean genotypes

**Fig. 2:**
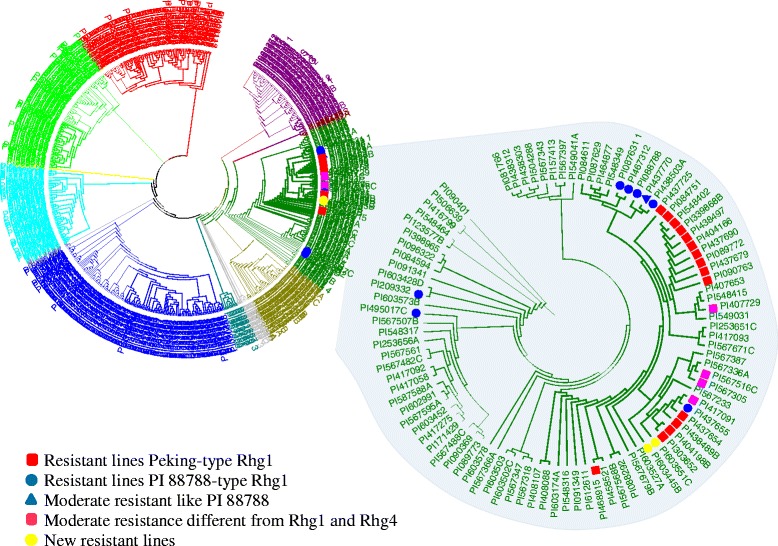
Phylogenetic tree showing distribution of nematode resistant genotypes (denoted with bullets) in a set of 553 soybean plant introduction accessions

**Fig. 3:**
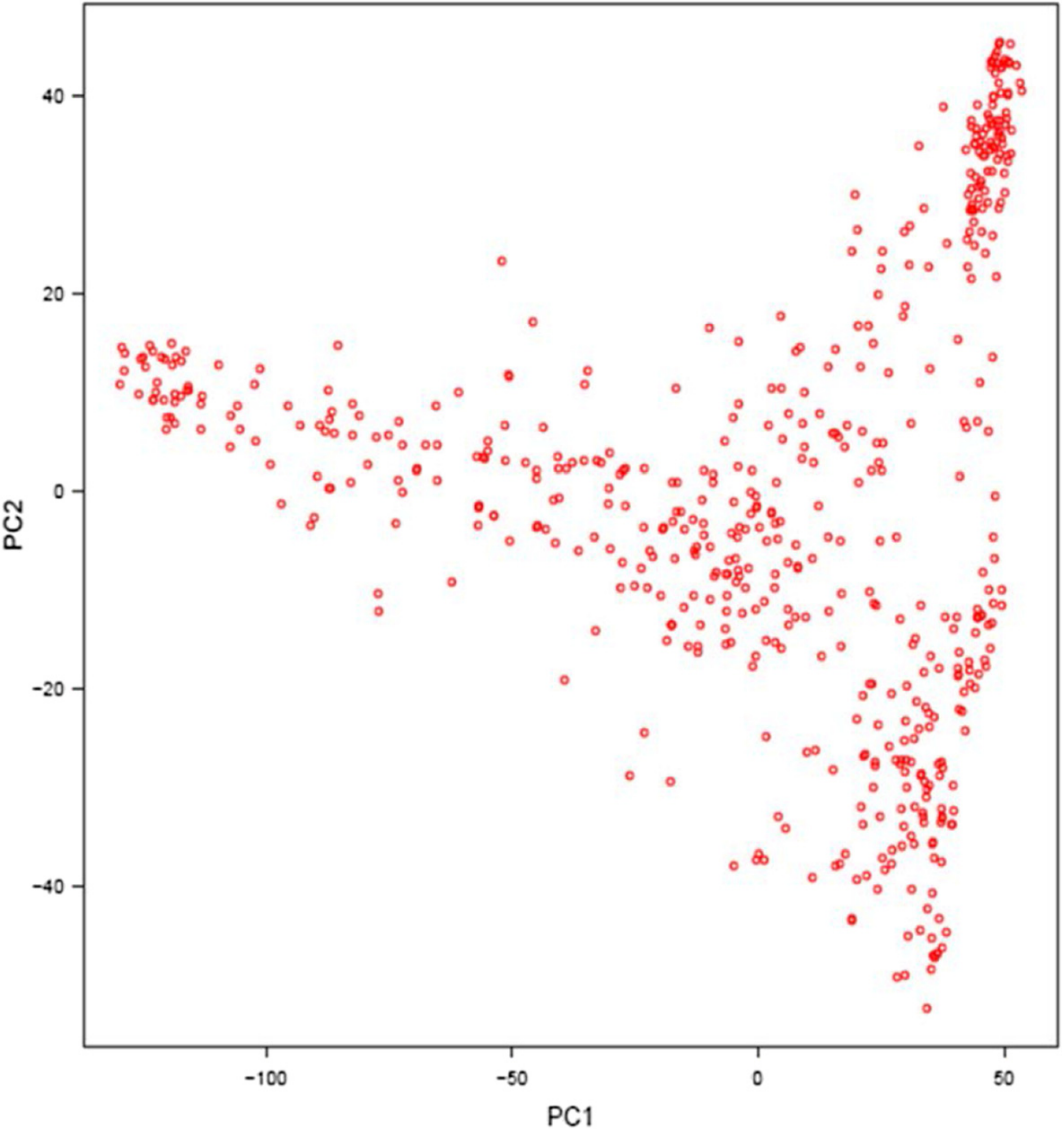
Principal Component Analysis (PCA) of a diverse set of 553 soybean plant introductions (PIs) genotyped with the SoySNP50K iSelect BeadChip data set

**Fig. 4:**
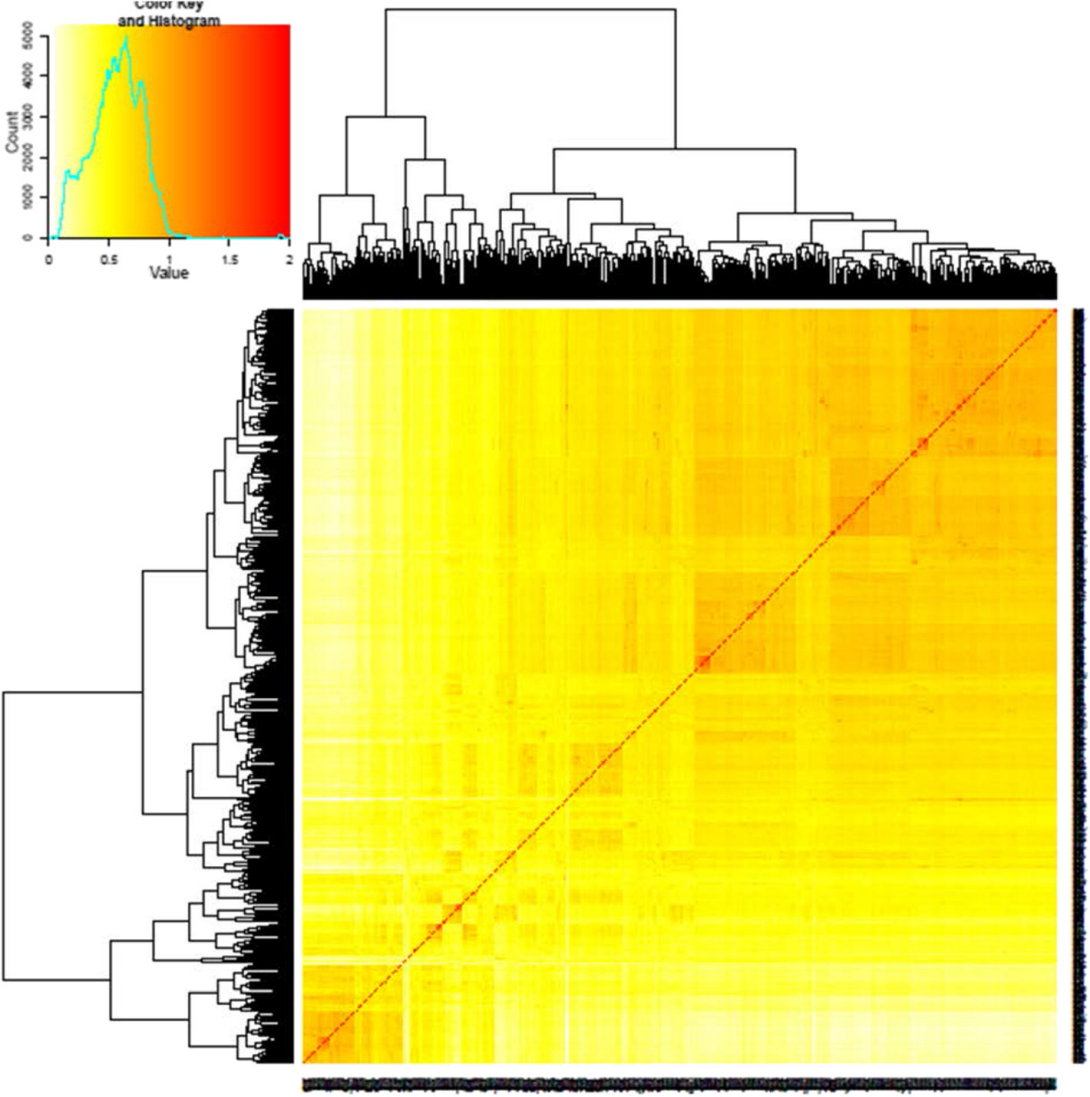
Kinship matrix among the diverse 553 soybean plant introductions (PIs) estimated using the SoySNP50K iSelect BeadChip data set

A neighbor-joining (NJ) tree for a set of 553 soybean accessions was constructed based on Nei’s genetic distance obtained from the TASSEL software with 35,270 SNPs and a graphical visualization of phylogenetic tree was made using MEGA5 software (Fig. 2). The resulting NJ tree showed five divergent subgroups and interestingly all the known resistant PIs belonged to the same sub-cluster. The sub-cluster has grouped genetically very similar genotypes even though these PIs do not show a further distinct grouping on the basis of resistance source. For instance, PI 437655, having PI 88788-type resistance, was grouped together with PI 437654 that has been well known for resistance source and commonly used to develop new SCN resistance soybean cultivars, including cv. Hartwig. Principal component analysis (PCA) also showed dispersed genotypes among different components suggesting very diverse genetic backgrounds (Fig. 3). Kinship analysis showed a clustering pattern of the PIs similar to the NJ tree. The PCA and Kinship information were further utilized for the association analysis (Fig. 4).

### Linkage disequilibrium (LD)

**Fig. 5:**
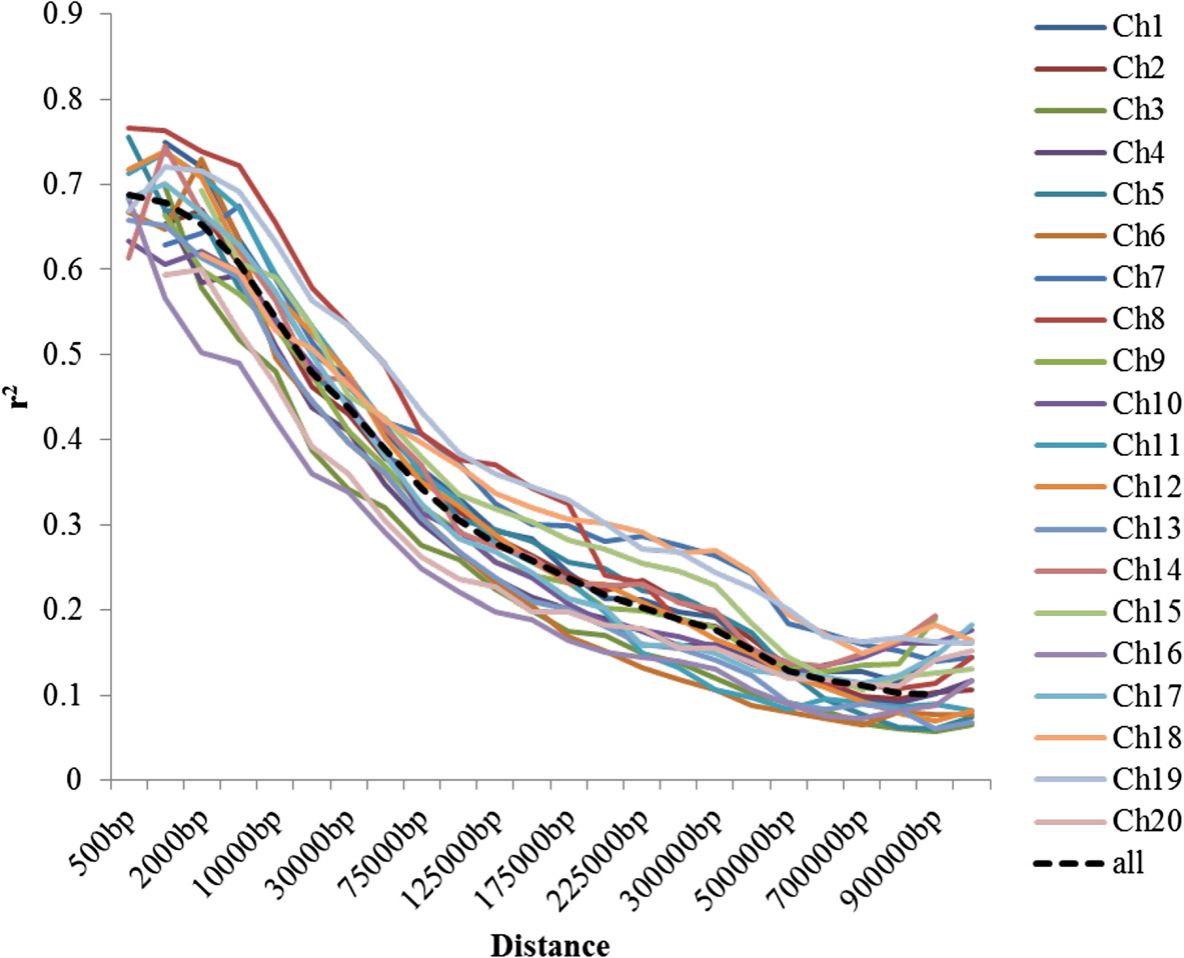
Linkage disequilibrium (LD) pattern across different soybean chromosomes showing negative relationship of distance between loci with *r* ^*2*^ values

The distribution of correlation coefficients (*r*^*2*^) between SNPs located at different physical distances of each chromosome was calculated to establish LD relationship among loci. As expected, the *r*^*2*^ value declined as the physical distance between the loci increased (Fig. 5). LD decay for each chromosome was different (Table 1). In addition, LD decay varied among all chromosomes, ranging from approximately 125 kb to 600 kb. The average LD decay for all chromosomes was estimated at approximately 250 kb, when the value of the cut off for *r*^*2*^ was set to 0.2. Since, soybean is a self-pollinated crop, a greater extent of LD is expected as compared to out-crossed crops, such as maize.

**Table 1:** Linkage disequilibrium (LD) decay estimated for different soybean chromosomes

Chromosome No.	Chr. size (Mb)	No. of markers*	LD decay (Kb)	Required marker**
1	55.92	1,426	250	224
2	51.66	2,130	300	172
3	47.78	1,428	150	319
4	49.24	1,677	200	246
5	41.94	1,513	300	140
6	50.72	1,595	175	290
7	44.68	1,801	500	89
8	47.00	2,167	250	188
9	46.84	1,649	250	187
10	50.97	1,846	200	255
11	39.17	1,433	200	196
12	40.11	1,271	250	160
13	44.41	2,240	200	222
14	49.71	1,677	300	166
15	50.94	2,130	400	127
16	37.40	1,496	125	299
17	41.91	1,734	225	186
18	62.31	2,894	500	125
19	50.59	1,855	600	84
20	46.77	1,308	150	312
Total	950.07	35,270	250	3,800

Chr. – Chromosome, LD – Linkage disequilibrium, *– number of markers used in present study, **– Average number of required makers was estimated based on the chromosome size and LD decay at *r* ^*2*^ = 0.2

### Genome-wide association study (GWAS)

**Fig. 6:**
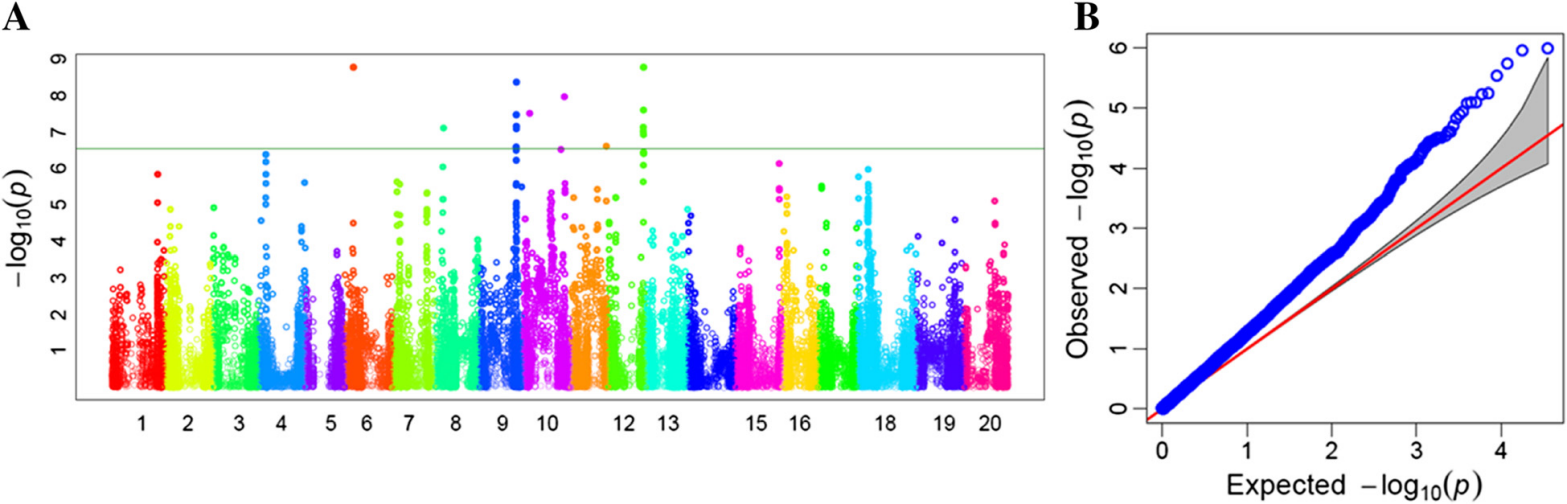
Significantly associated single nucleotide polymorphism (SNP) with SCN resistance in soybean identified by genome-wide association study (GWAS). **a** Manhattan plot; **b** Q-Q plot using generalized linear model (GLM)

**Fig. 7:**
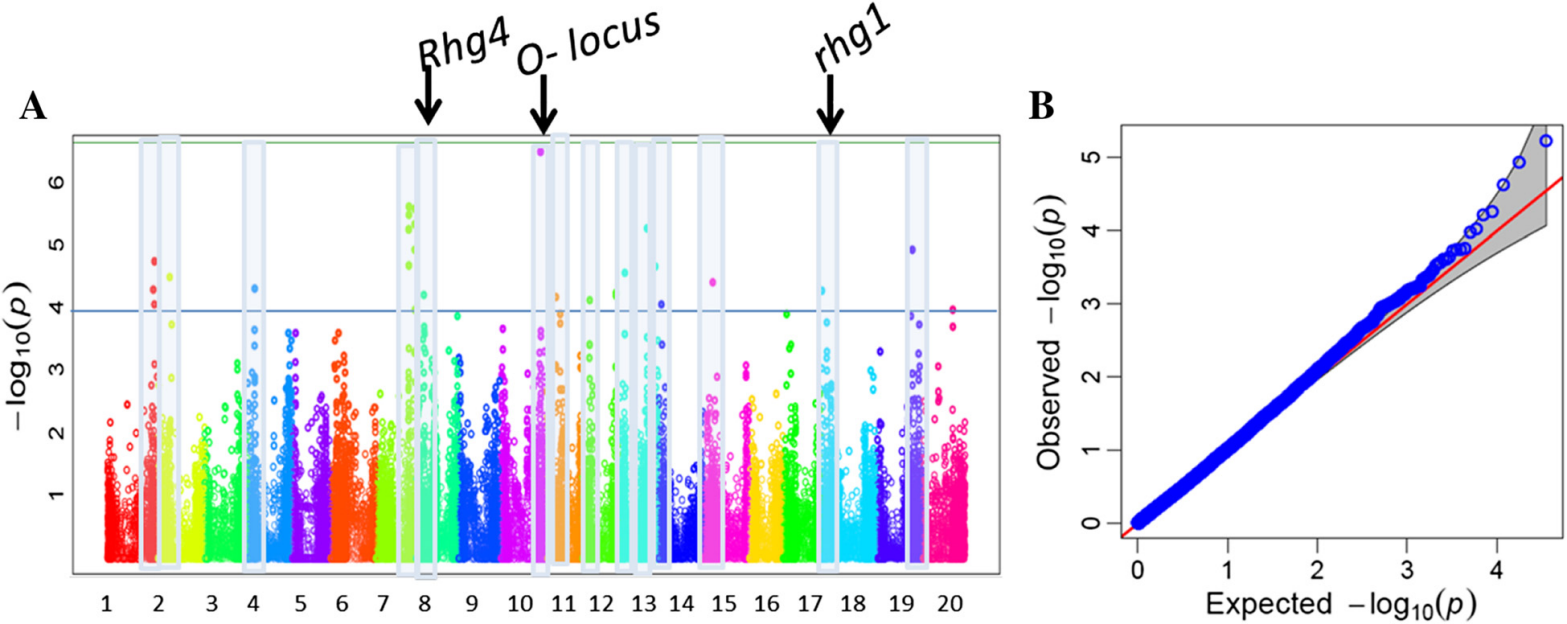
Significantly associated single nucleotide polymorphism (SNP) with SCN resistance in soybean identified by genome-wide association study (GWAS). A) Manhattan plot; B) Q-Q plot using mixed linear model (MLM)

GWAS was performed using generalized linear model (GLM) identified 223 SNPs distributed over 19 different chromosomes and associated with resistance to SCN HG Type 0 (Fig. 6a). These SNPs represent a minimum allele frequency (MAF) ranging from 0.05 to 0.45 and with a highest *p* value of 1.7E-9.0. A Q-Q plot representing expected and observed probability of getting association of SNPs with a phenotype showed possibility of high number of false positive associations (Fig. 6b). Therefore, a mixed linear model (MLM), which is one of the most effective methods for controlling false positives in GWAS, was used for further analysis. The efficient mixed-model association (EMMA) model was used in the analysis to correct for confounding effects due to subpopulation structure and relatedness between individuals. The MLM identified 41 SNPs distributed over 16 loci on 14 different chromosomes that were significantly associated with resistance to SCN HG Type 0 (Table 2, Fig. 7). The genomic region on Chr. 10 showed a higher peak level of significance (*p*-value = 3.29E-07, 2.42E-06) comprising two SNPs. The most significant SNP on Chr. 10 showed 51 % phenotypic variation, significantly higher than the variation (47 %) estimated without the SNP (Table 2). On Chr. 7, two loci at 36.5 Mb and 43.0 Mb comprising five and four SNPs, respectively, were found to be associated with SCN resistance. The most significant SNP at these loci contributed 50 % phenotypic variation. Interestingly, the known loci *rhg1* on Chr. 18 and *Rhg4* on Chr. 8, were also identified in this study. Highly significant SNPs on Chr. 8 between 7.5 to 8.6 Mb and on Chr. 18 between 1.2 to 6.6 Mb were associated with SCN resistance. These loci did not show a high level of significance even though these loci harbor a very high level of resistance. Since the number of genotypes carrying the resistance allele for either of these genes was very few which affects the level of significance in GWAS.

**Table 2:** Details of loci governing SCN resistance in soybean identified by genome-wide association study performed using a set of diverse soybean plant introductions (PIs) genotyped with the SoySNP50K iSelect BeadChip

Chromosome no.	MSS position	MSS P-value	R^2^*	R^2^**	Total SNPs	Significant locus	
						Start	End
1	51957108	1.85E-05	0.47	0.50	3	50726668	51960351
2	13017725	3.29E-05	0.47	0.49	1	13663384	13663384
4	7627278	4.94E-05	0.47	0.49	1	7627278	7627278
7	36560926	2.42E-06	0.47	0.50	5	36480188	36560926
7	43095766	2.63E-06	0.47	0.50	4	43093289	43095766
8	8607787	6.36E-05	0.47	0.49	2	7571195	8607787
10	43812212	3.29E-07	0.47	0.51	2	40113201	43812212
11	10174912	6.88E-05	0.47	0.49	3	10174912	14827458
12	37554204	5.86E-05	0.47	0.49	2	37537049	37554204
13	6988591	2.85E-05	0.47	0.49	8	29081	105760
13	31806761	5.52E-06	0.47	0.50	2	31806761	31828223
14	3172907	9.06E-05	0.47	0.49	1	3172907	3172907
15	10320348	3.93E-05	0.47	0.49	1	10320348	10320348
18	1286527	5.46E-05	0.47	0.49	2	1286527	6646067
19	39002612	1.18E-05	0.47	0.50	3	37531417	46497770
20	33218932	0.0001	0.47	0.49	1	33218932	33218932

Chr. – Chromosome, MSS – Most significant SNP, *– R square of model without SNP, **– R square of model with SNP

### Candidate genes for SCN resistance at GWAS loci

Annotation information of the soybean genome sequence assembly suggested 2,352 genes at the 16 GWAS loci identified in the present study. Functional categorization of genes based on gene ontology showed the highest number of genes involved in transcription factor/DNA binding activity followed by catalytic activity in the molecular function category (Additional file 2: Figure S1). Furthermore, information of significantly over-represented gene ontology (GO) categories was used for sorting the list as per priority. A total of 158 resistance gene analogs (RGA) and disease resistance genes were identified (Additional file 3: Table S2). Out of these, only 106 genes were observed to be expressed in RNA-seq data available for 14 different soybean tissues. Analysis of microarray experimental data available for SCN (*H. glycine*) infected cells captured using laser micro-dissection (E-MEXP-876) and infected root tissue (E-MEXP-808) showed differential expression for most of the candidate genes [53, 54] (Additional file 4: Table S3). The GWAS loci identified on Chr. 8 and Chr. 18 showed a presence of the previously known and well characterized *rhg1* and *Rhg4* genes, respectively.

### GWAS for simple and moderately complex traits

**Fig. 8:**
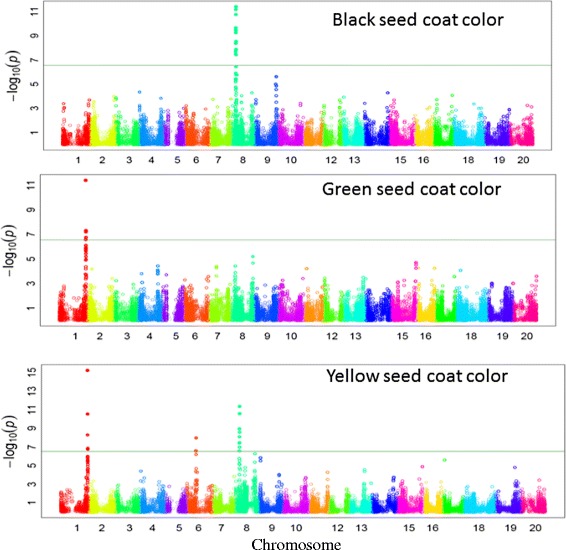
Significantly associated single nucleotide polymorphism (SNP) with different seed coat colors in soybean identified by genome-wide association study (GWAS)

A total of nine significant GWAS loci were identified for four simple traits, including seed coat color, flower color, pubescence color, and stem growth habit (Table 3). For seed coat color, different loci were observed to govern different colors. For instance, black, green, and brown seed coat colors are governed by loci on Chrs. 08, 01, and 15, respectively. Yellow seed coat color, which is more common in soybean cultivars, was observed to be governed by loci on three different chromosomes. The loci on Chrs. 01 and 08 for green and black seed coat color were also found to be significantly associated with yellow seed coat. In addition, locus on Chr. 06 was found to be associated with yellow seed coat color (Fig. 8).

**Table 3:** Details of loci governing four simple and two moderately complex inherited traits in soybean identified by genome-wide association study performed using a diverse set of 553 soybean plant introductions (PIs) genotyped with the SoySNP50K iSelect BeadChip data set

Trait	Chromosome no.	MSS position	MSS P-value	R^2^*	R^2^**	Total SNPs	Significant locus	
							Start	End
Seed coat color								
Black	8	8427110	3.89E-12	0.58	0.62	19	7780581	8627848
Green	1	52253980	3.99E-12	0.24	0.31	7	52253980	52743661
Brown	15	12772149	5.70E-07	0.38	0.41	1	12772149	12772149
Yellow	1	52253980	5.80E-16	0.52	0.58	6	52249479	52717757
	8	8462762	4.40E-12	0.52	0.56	10	8227016	8627848
	6	18766611	1.04E-08	0.52	0.55	5	18118558	18766611
Flower color	13	4559799	3.13E-41	0.40	0.63	90	2493212	5074933
Pubescence color	6	18766611	8.01E-28	0.38	0.53	22	17567713	18810733
Stem growth habit	19	45000827	2.71E-31	0.38	0.56	40	44367612	45325838
Pod shattering	16	29242023	1.37E-07	0.26	0.30	6	29215338	29666971
Lodging	19	45000827	5.10E-14	0.23	0.32	11	44734359	45178132

Chr. – Chromosome, MSS – Most significant SNP, *– R square of model without SNP,** – R square of model with SNP

Additionally, GWAS precisely identified the same locus (*W1*) on Chr. 13, which has been previously identified by Zabala and Vodkin [38] and Sonah et al. [33] (Additional file 5: Figure S2). Similarly, for pubescence, a previously known locus on Chr. 06, was confirmed [33]. A GWAS locus identified for stem growth habit on Chr. 19 with a high level of significance was co-located with the previously known loci Dt1 [39].

**Fig. 9:**
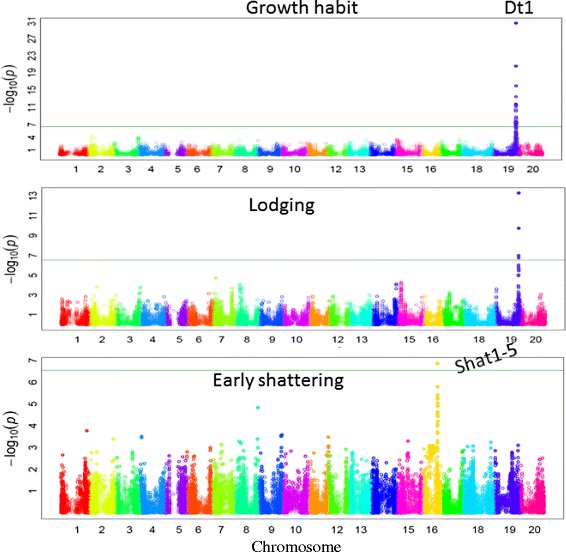
Significantly associated single nucleotide polymorphism (SNP) with growth habit, lodging and, early shattering in soybean identified by genome-wide association study (GWAS)

A GWAS locus for a moderately complex trait, like plant lodging, was identified on Chr. 19. It was exactly the same locus identified for stem growth habit. It is known that non-determinacy is associated with lodging and this might be the reason for co-localization of GWAS loci. For another moderately complex trait, like pod shattering, a significant GWAS locus was identified on Chr. 16, which has been recently identified by Dong et al. [40] (Fig. 9).

GWAS loci identified for four simple traits and two moderately complex traits confirm the known loci, which raised the confidence level of this study.

## Discussion

### Phylogenetic variation for SCN resistance in soybean

Most of the resistant PI lines identified in this study were grouped together in a phylogenetic tree, suggesting a common progenitor. Even though the resistant PI lines resemble a very similar genetic background, they carry different resistance sources, like Peking-type and PI 88788-types. This may be due to the historic breeding activities or these resistance sources may have evolved very recently. PI 437654 and PI 437655 are genetically very similar, but carry different types of resistance (Fig. 2). The cultivar Hartwig has been developed by using PI 437654. New resistance PIs clustering along with known resistant PIs most probably carry the same type of resistance. Previously, many efforts have been made to understand genetic divergence between North American ancestral soybean lines and SCN resistance PI lines using chloroplast specific SSR markers [41]. Another effort has analyzed genetic diversity of soybean and the established a core collection focused on resistance to soybean cyst nematode [42]. Both studies have been performed using a limited set of markers; therefore, the genetic relatedness was not well defined. In the present study, the genetic distance estimated using SoySNP50K genotyping was more robust and helpful to define the population structure.

### Linkage disequilibrium decay in the soybean genome

Many different factors, such as natural selection, domestication, founding events, genetic diversity, and population stratification, affect the extent of LD [31, 36]. Loci governing domesticated traits, like seed size, seed color, and flowering, have showed longer LD decay [31, 36]. Highest LD was observed on Chr. 19, which harbors the E3 locus known for flowering time [43]. Compared to maize (2 – 50 kb) and barley, longer LD decay was observed in soybean (Table 1). This is due to the self-pollination fertilization nature of soybean (125–600 kb) even though it is longer than the other self-pollinated crops, like rice (75–150 kb). Similar results for LD decay in soybean was observed in previous studies [44, 45]. Soybean has very narrow genetic diversity compared other cultivated crops. The genetic bottle-neck has increased LD block-size resulting into longer GWAS regions being associated with the phenotype (Table 2). Because of the longer LD, a relatively less number of markers is required for the effective GWAS in soybean (Table 1). However, the LD decay varied at different loci and chromosomes [31]. Therefore, a higher number of markers than what is estimated is required to ensure coverage across all the LD blocks.

### Genetic architecture of SCN resistance in soybean

Besides the recent identification of two major genes *rhg1* and *Rhg4*, very little has been known about the resistance mechanism involved in SCN resistance and genetic variation that exists in the soybean germplasm. The host-pathogen interaction is very complex involving multiple genes, which trigger the molecular signaling and subsequent responses. The identification of loci governing resistance not only helps the genetic improvement of cultivars but also facilitates the identification of genes and the understanding of molecular mechanisms involved in the resistance process. The 16 GWAS loci for SCN resistance identified in the present study provided the molecular basis to understand the variable genetic responses observed in soybean germplasm. Apart from the conventional QTL mapping, which was typically based on a segregation of resistance in narrow genetic backgrounds, GWAS captures simultaneously the vast genetic variation existing in soybean germplasm. Most of the GWAS loci confirmed the previously identified QTL for SCN resistance (Additional file 6: Table S4). Of these, a novel QTL recently identified on Chr. 10 [4] was also observed with this GWAS. We believe that these are the major 16 loci which define the genetic architecture of SCN resistance in soybean.

## Conclusions

In the present study, we report the identification and confirmation of QTL significantly associated with resistance to SCN HG Type 0 (race 3) in a diverse panel of 553 soybean germplasm accessions in maturity groups from III to V. It included the known QTL, such as the *rhg1* and *Rhg4*, and also novel QTL, such as Chr. 10-QTL, which was recently reported. GWAS using the appropriate analysis model enabled us to identify several SNP markers significantly associated with QTL. The availability and accessibility of the reference soybean genome sequence and gene annotation also facilitated the identification of candidate genes, leading to the functionality analysis. The results showed that GWAS can be employed as an effective strategy for identifying complex traits in soybean and for narrowing GWAS-defined genomic regions, which facilitates positional cloning of the causal gene(s).

## Materials and methods

### Plant materials

The germplasm analyzed included 553 accessions in maturity groups III to V (Additional file 7: Table S5). When these accessions were selected, the core collection was being formed for the USDA Soybean Germplasm Collection but was not yet completed. The procedures used to select the core collection were used to select these accessions and 95 % of the lines used in this research were included in the final core collection [46]. The entries analyzed here represent approximately 70 % [3] of accessions in core collection in each of three maturity groups included. The core collection contains approximately 10 % of the total number of introduced soybean accessions in the USDA Soybean Germplasm Collection. Selection of accessions for the core collection was made using origin, qualitative, and quantitative data. Accessions were divided in groups based on origin and then further subdivided based on maturity group, which classifies soybean accessions based on photoperiod and temperature response. A multivariate proportional sampling strategy within each stratum was determined to be the optimal procedure for identifying a sample of accessions that best represents the diversity of the total collection [46].

### Phenotyping for soybean cyst nematode (SCN) and other traits

Greenhouse bioassays of a collection of 553 soybean accessions was conducted in the SCN phenotyping facility at the University of Missouri in Columbia, Missouri, following the established procedure described by Arelli et al. [47] and Vuong et al. [3]. Briefly, five 5-day soybean seedlings of each accession and seven indicator lines, PI 548402 (Peking), PI 88788, PI 90763, PI 437654, PI 209332, PI 89772, and PI 548316, were inoculated with 2,000 ± 25 eggs of HG Type 0, corresponding to race 3. A SCN susceptible cultivar, Hutcheson, was used as a check to evaluate the response to a nematode population. Two greenhouse bioassays were independently carried out. The experiments were maintained at 27 ± 1 °C and watered daily. Thirty days post inoculation, nematode cysts were washed from roots of each plant and counted using a fluorescence-based imaging system [48]. The female index (FI) estimation was used as described in the following formula:

$$ \mathrm{F}\mathrm{I} = \left(\begin{array}{l}\mathrm{a}\mathrm{v}e\mathrm{r}\mathrm{a}\mathrm{g}e\ n\mathrm{u}\mathrm{m}\mathrm{b}e\mathrm{r}\ \mathrm{o}\mathrm{f}\ \mathrm{f}e\mathrm{m}\mathrm{a}\mathrm{l}e\ \mathrm{c}\mathrm{yst}\ ne\mathrm{m}\mathrm{a}\mathrm{t}\mathrm{o}\mathrm{d}e\mathrm{s}\ \mathrm{o}n\ \mathrm{a}\ \mathrm{t}e\mathrm{s}\mathrm{t}\ \mathrm{soyb}e\mathrm{a}n\ \mathrm{l}\mathrm{i}ne\ /\ \mathrm{a}\mathrm{v}e\mathrm{r}\mathrm{a}\mathrm{g}e\ \\ {}n\mathrm{u}\mathrm{m}\mathrm{b}e\mathrm{r}\ \mathrm{o}\mathrm{f}\ \mathrm{f}e\mathrm{m}\mathrm{a}\mathrm{l}e\ ne\mathrm{m}\mathrm{a}\mathrm{t}\mathrm{o}\mathrm{d}e\mathrm{s}\ \mathrm{o}n\ \mathrm{t}\mathrm{h}e\ \mathrm{susc}e\mathrm{ptibl}e\ \mathrm{c}\mathrm{h}e\mathrm{c}\mathrm{k}\end{array}\right)\times 100 $$

Phenotyping for other traits including seed coat color, flower color, pubescence color, stem growth habit, lodging, and pod shattering was performed under field conditions.

### Genotyping data of 50 K SNP array

Over 50,000 SNP markers of the soybean genome generated in the SoySNP50K iSelect BeadChip [49] were accessed from the soybean database (*http//**www.soybase.org*). A total of 35,270 SNPs were selected for GWAS after excluding SNPs with more than 20 % missing data and a minor allele frequency less than 5 %.

### Genome-wide association study (GWAS)

All GWAS analyses were performed using TASSEL 4.0 and the Genomic Association and Prediction Integrated Tool (GAPIT) [50, 51]. A kinship matrix (K) was calculated using the VanRaden method and EMMA method to determine relatedness among individuals [52, 53]. The general linear model (GLM) included the principle component analysis (PCA) model, and a model that did not control for PCA was tested for analysis. The mixed linear model (MLM) used in this study comprised the K model, and the PCA + K model. A compressed mixed linear models (CMLM) incorporating a matrix K along with PCA was also used. In this study, negative log (1/n) was used as a threshold since the Bonferroni test (0.05/numbers of samples) criterion is typically too strict to be a threshold. The statistical threshold for GWAS was decreased to obtain the true associations in plants. The estimates of the LD were determined using the squared allele-frequency correlations (*r*^*2*^) for pairs of loci, calculated using software TASSEL3.0 [51].

### Candidate genes of SCN resistance

Genomic sequence along with information of predicted genes around the most significant GWAS loci were retrieved from the Phytozome database [54]. Candidate gene search was performed with the predicted gene models at around 0.5 Mb flanking to the significant GWAS loci. Functional annotation of the genes was performed using the BLAST2GO tool with BLASTx and BLASTp search [55] and the SoyKB database [56]. Kyoto encyclopedia of genes and genomes (KEGG) was used to predict pathways for candidate genes [57]. Microarray expression profiling of SCN infected root cells captured by laser assisted micro-dissection and root tissue at different time periods after inoculation were analyzed using the Genevestigator package [www.genevestigator.com, [58, 59].

## Additional files

Additional file 1: Table S1. Plant introductions were characterized as known and novel sources of resistance to soybean cyst nematode in this study. (DOCX 17 kb) Additional file 2: Figure S1. Gene ontology characterization of candidate genes at significant loci associated with SCN resistance. (DOCX 150 kb) Additional file 3: Table S2. Functional annotation of candidate genes located at significant GWAS loci for SCN resistance in soybean. (DOCX 24 kb) Additional file 4: Table S3. Differential expression pattern of candidate genes evaluated in *Heterodera glycines* infected cells captured by laser micro-dissection & root tissue. (DOCX 28 kb) Additional file 5: Figure S2. Significantly associated SNPs with the flower color in soybean identified by genome-wide association study (GWAS). (DOCX 635 kb) Additional file 6: Table S4. A comparison of genome-wide association study (GWAS) loci identified in the present study and quantitative trait loci (QTL) previously reported for resistance to soybean cyst nematode (SCN) from different sources of resistance showing the consistency of genetic locations of GWAS and QTL analysis. (DOCX 13 kb) Additional file 7: Table S5. Five hundred fifty three soybean germplasm accessions with morphological characteristics, agronomic traits, and code definitions were utilized in genome-wide association study. (DOCX 61 kb)

## Abbreviations

GWAS: Genome-wide association mapping
SCN: Soybean cyst nematode
QTL: Quantitative trait loci
SSR: Simple sequence repeat
SNPs: Single nucleotide polymorphism
LD: Linkage disequilibrium
GLM: General linear model
MLM: Mixed linear model
