# Supplementary material for: Genetic architecture of cyst nematode resistance revealed by genome-wide association study in soybean
Source: BMC Genomics. 2015 Aug 12;16:593. doi: 10.1186/s12864-015-1811-y (PMC4533770; doi:10.1186/s12864-015-1811-y)
Supplement: Additional file 1: Table S1. — Plant introductions were characterized as known and novel sources of resistance to soybean cyst nematode in this study. (DOCX 17 kb) [file 12864_2015_1811_MOESM1_ESM.docx]

**Table S1**. Plant introductions were characterized as known and novel sources of resistance to soybean cyst nematode in this study.

| **No** | **PI** | **Origin** | **MG** | **Growth** |
| --- | --- | --- | --- | --- |
| **Known Source of Resistance** | | | | |
| 1 | PI084751 | Unknown | IV | Determinant |
| 2 | PI087631-1 | Unknown | III | Indeterminant |
| 3 | PI088788 | China | III | Indeterminant |
| 4 | PI089772 | China | IV | Indeterminant |
| 5 | PI090763 | China | IV | Indeterminant |
| 6 | PI303652 | China | V | Indeterminant |
| 7 | PI339868B | South Korea | IV | Determinant |
| 8 | PI404166 | China | III | Indeterminant |
| 9 | PI404198B | China | IV | Indeterminant |
| 10 | PI407729 | China | IV | Indeterminant |
| 11 | PI437654 | China | III | Indeterminant |
| 12 | PI437655 | China | III | Indeterminant |
| 13 | PI437679 | China | IV | Semi-determinant |
| 14 | PI437690 | China | III | Semi-determinant |
| 15 | PI437725 | China | IV | Semi-determinant |
| 16 | PI437770 | China | III | Indeterminant |
| 17 | PI438489B | Russia | IV | Indeterminant |
| 18 | PI438497 | Russia | III | Determinant |
| 19 | PI468915 | China | II | Indeterminant |
| 20 | PI548349 | North Korea | III | Indeterminant |
| 21 | PI548402 | China | IV | Determinant |
| 22 | PI549031 | China | III | Indeterminant |
| 23 | PI567305 | China | IV | Indeterminant |
| 24 | PI567387 | China | IV | Indeterminant |
| 25 | PI567516C | China | IV | Indeterminant |
| 26 | PI612611 | North Korea | III | Indeterminant |
| **Novel Resistance Source** | | | | |
| 1 | PI461509 | China | I | Indeterminant |
| 2 | PI603587A | China | I | Indeterminant |
| 3 | PI091102 | China | II | Indeterminant |
| 4 | PI416762 | Japan | II | Indeterminant |
| 5 | PI417091 | Japan | II | Indeterminant |
| 6 | PI438503A | Russia | II | Indeterminant |
| 7 | PI458520 | China | II | Indeterminant |
| 8 | PI467312 | China | II | Indeterminant |
| 9 | PI089008 | China | II | Indeterminant |
| 10 | PI467332 | China | II | Indeterminant |
