# Supplementary material for: Genetic architecture of cyst nematode resistance revealed by genome-wide association study in soybean
Source: BMC Genomics. 2015 Aug 12;16:593. doi: 10.1186/s12864-015-1811-y (PMC4533770; doi:10.1186/s12864-015-1811-y)
Supplement: Additional file 2: Figure S1. — Gene ontology characterization of candidate genes at significant loci associated with SCN resistance. (DOCX 150 kb) [file 12864_2015_1811_MOESM2_ESM.docx]

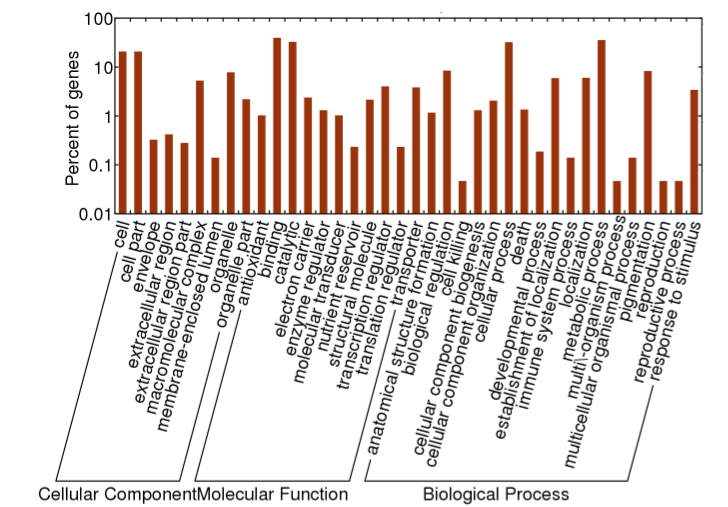


**Figure S1** Gene ontology characterization of candidate genes at significant loci associated with soybean cyst nematode resistance.
