# Supplementary material for: Genetic architecture of cyst nematode resistance revealed by genome-wide association study in soybean
Source: BMC Genomics. 2015 Aug 12;16:593. doi: 10.1186/s12864-015-1811-y (PMC4533770; doi:10.1186/s12864-015-1811-y)
Supplement: Additional file 3: Table S2. — Functional annotation of candidate genes located at significant GWAS loci for SCN resistance in soybean. (DOCX 24 kb) [file 12864_2015_1811_MOESM3_ESM.docx]

**Table S2** Functional annotation of candidate genes located at significant GWAS loci for SCN resistance in soybean.

| Gene_ID | Pfam_Id | Functional Annotation | KOG Annotation |
| --- | --- | --- | --- |
| Glyma01g38920 | PF00069 PF00008 | LRR Receptor-Like Protein Kinase | Ser/Thr protein kinase |
| Glyma01g39000 | PF00931 PF05659 PF00560 | LRR -Containing Protein Disease Resistance Protein (CC-NBS-LRR Class), | Apoptotic Atpase |
| Glyma01g39010 | PF05659 PF00931 | Disease Resistance Protein (CC-NBS-LRR Class), LRR -Containing Protein | Apoptotic Atpase |
| Glyma01g39020 | PF00069 | Serine/Threonine Kinase | Ser/Thr protein kinase |
| Glyma01g39071 | PF00069 | Mitogen-Activated Kinase Kinase Kinase | Mekk & Related Ser/Thr protein kinases |
| Glyma01g39380 | PF00069 | Mitogen-Activated Kinase Kinase Kinase | Mekk & Related Ser/Thr protein kinases |
| Glyma01g39420 | PF00069 | LRR Receptor-Like Protein Kinase | Ser/Thr protein kinase |
| Glyma01g39451 | PF00010 | Basic Helix-Loop-Helix/Leucine Zipper Transcription Factor Basic Helix-Loop-Helix (Bhlh) | |
| Glyma01g39470 |  | F-Box/LRR Protein | |
| Glyma01g39660 | PF00646 | F-Box/LRR Protein | LRR Proteins, Some Proteins Contain F-Box |
| Glyma04g09070 | PF00069 | Casein Kinase-Related | Casein Kinase (Serine/Threonine/Tyrosine Protein Kinase) |
| Glyma04g09080 | PF00069 | Casein Kinase-Related Subfamily Not Named | Casein Kinase (Serine/Threonine/Tyrosine Protein Kinase) |
| Glyma04g09160 | PF00560 PF00069 PF08263 | LRR Receptor-Like Protein Kinase | Ser/Thr protein kinase |
| Glyma04g09210 | PF00069 | Serine/THREONINE-PROTEIN KINASE IAL-RELATED | Ser/Thr protein kinase |
| Glyma04g09380 | PF00560 PF00069 PF08263 | LRR Receptor-Like Protein Kinase | Ser/Thr protein kinase |
| Glyma04g09610 | PF03822 PF00069 | Serine/Threonine-Protein Kinase CDS1 | Ser/Thr protein kinase |
| Glyma07g31140 | PF00560 PF07714 | LRR Receptor-Like Protein Kinase | Ser/Thr protein kinase |
| Glyma07g31200 | PF00450 | Serine CARBOXYPEPTIDASE II | Serine Carboxypeptidases (Lysosomal Cathepsin A) |
| Glyma07g31460 | PF07714 | LRR Receptor-Like Protein Kinase | Ser/Thr protein kinase |
| Glyma07g31970 | PF00560 PF11721 | LRR Receptor-Like Protein Kinase | |
| Glyma07g38410 | PF00481 | Protein Phosphatase 2c Subfamily Not Named | Serine/Threonine Protein Phosphatase |
| Glyma07g38445 |  | F-Box/LRR Protein | |
| Glyma08g10470 | PF00069 | Serine/THREONINE KINASE | Ser/Thr protein kinase |
| Glyma08g10640 | PF07714 | LRR Receptor-Like Protein Kinase | Ser/Thr protein kinase |
| Glyma08g10680 |  | F-Box/LRR Protein Os04g0485800 Protein | |
| Glyma08g11350 | PF00069 PF00560 PF08263 | LRR Receptor-Like Protein Kinase | Ser/Thr protein kinase |
| Glyma08g11370 | PF03447 PF01842 PF00742 PF00696 | Aspartate Kinase | Homoserine Dehydrogenase |
| Glyma08g11490 | PF00464 | Serine HYDROXYMETHYLTRANSFERASE | Glycine/Serine Hydroxymethyltransferase |
| Glyma08g11590 | PF00076 PF00630 | Family Not Named Nuclear Protein-Like ( Uncharacterized Protein) (Os01g0101600 Protein) | Splicing Factor, Arginine/Serine-Rich |
| Glyma10g31630 | PF00069 | Mitogen-Activated Kinase Kinase Kinase | |
| Glyma10g32090 | PF08263 PF07714 PF00560 | LRR Receptor-Like Protein Kinase | Ser/Thr protein kinase |
| Glyma10g32280 | PF03822 PF00069 | Serine/THREONINE KINASE | Ser/Thr protein kinase |
| Glyma10g32490 | PF00069 | Casein Kinase Casein Kinase-Related | Casein Kinase (Serine/Threonine/Tyrosine Protein Kinase) |
| Glyma10g32785 | PF01582 PF00931 PF00560 | LRR -Containing Protein Subfamily Not Named | |
| Glyma10g32800 | PF01582 PF00560 PF00931 | LRR -Containing Protein Subfamily Not Named | Apoptotic Atpase |
| Glyma10g33040 | PF00149 | Serine/THREONINE PROTEIN PHOSPHATASE SUBFAMILY NOT NAMED | Serine/Threonine Specific Protein Phosphatase PP1, Catalytic Subunit |
| Glyma10g33970 | PF08263 PF00069 PF00560 | LRR Receptor-Like Protein Kinase | Ser/Thr protein kinase |
| Glyma10g34061 | PF00931 | LRR -Containing Protein | Apoptotic Atpase |
| Glyma10g35120 | PF00450 | Serine PROTEASE FAMILY S10 Serine CARBOXYPEPTIDASE | Serine Carboxypeptidases (Lysosomal Cathepsin A) |
| Glyma11g14240 | PF07714 | LRR Receptor-Like Protein Kinase | Ser/Thr protein kinase |
| Glyma11g15490 | PF11721 PF07714 | LRR Receptor-Like Protein Kinase | Ser/Thr protein kinase |
| Glyma11g15550 | PF00069 | LRR Receptor-Like Protein Kinase | Ser/Thr protein kinase |
| Glyma11g16303 |  | F-Box/LRR Protein | |
| Glyma11g17081 | PF07714 | LRR Receptor-Like Protein Kinase | |
| Glyma11g17120 | PF00010 | Basic Helix-Loop-Helix/Leucine Zipper Transcription Factor Basic Helix-Loop-Helix (Bhlh) Family Protein | |
| Glyma11g17875 | PF00931 | LRR -Containing Protein Subfamily Not Named | Apoptotic Atpase |
| Glyma11g18090 | PF07646 PF00149 | Serine/THREONINE PROTEIN PHOSPHATASE | Serine/Threonine Specific Protein Phosphatase PP1, Catalytic Subunit |
| Glyma12g33930 | PF00069 | LRR Receptor-Like Protein Kinase | Ser/Thr protein kinase |
| Glyma12g34410 | PF07714 | LRR Receptor-Like Protein Kinase | Ser/Thr protein kinase |
| Glyma12g34595 | PF07714 | Leucine-Rich Receptor-Like Protein Kinase LRR Receptor-Like Protein Kinase | |
| Glyma12g34680.1 | PF12609 | Wound-inducible protein |  |
| Glyma12g34690 | PF00931 | LRR -Containing Protein Subfamily Not Named | Apoptotic Atpase |
| Glyma12g34890 | PF11721 PF07714 | LRR Receptor-Like Protein Kinase | |
| Glyma13g00370 | PF00069 | LRR Receptor-Like Protein Kinase | Ser/Thr protein kinase |
| Glyma13g28370 | PF07714 | LRR Receptor-Like Protein Kinase | Ser/Thr protein kinase |
| Glyma13g28570 | PF00069 | Mapkk/Mekk | Serine-Threonine Protein Kinase Fused |
| Glyma13g28730 | PF00069 | LRR Receptor-Like Protein Kinase | Ser/Thr protein kinase |
| Glyma13g29190 | PF00069 | Ribosomal Protein S6 Kinase | Ser/Thr protein kinase |
| Glyma13g29200 | PF00646 | F-Box/LRR Protein | |
| Glyma13g29370 | PF00450 | Serine PROTEASE FAMILY S10 Serine CARBOXYPEPTIDASE Serine CARBOXYPEPTIDASE I (PLANTS) | Serine Carboxypeptidases (Lysosomal Cathepsin A) |
| Glyma13g29410 | PF00464 | Serine HYDROXYMETHYLTRANSFERASE | Glycine/Serine Hydroxymethyltransferase |
| Glyma14g04420 | PF00069 | LRR Receptor-Like Protein Kinase | Ser/Thr protein kinase |
| Glyma14g04433 | PF03822 PF00069 | Serine/THREONINE-PROTEIN KINASE PLK Serine/THREONINE-PROTEIN KINASE PLK1 | Ser/Thr protein kinase |
| Glyma14g04520 | PF00560 PF08263 | LRR Receptor-Like Protein Kinase | |
| Glyma14g04620 | PF00560 PF08263 | LRR Receptor-Like Protein Kinase | LRR Protein |
| Glyma14g04640 | PF00560 PF08263 | LRR Receptor-Like Protein Kinase | LRR Protein |
| Glyma14g04731 | PF00560 | LRR Receptor-Like Protein Kinase | LRR Protein |
| Glyma14g04750 | PF08263 PF00560 | LRR Receptor-Like Protein Kinase | LRR Protein |
| Glyma14g05040 | PF00560 PF08263 | LRR Receptor-Like Protein Kinase | LRR Protein |
| Glyma14g05060 | PF07714 | LRR Receptor-Like Protein Kinase | Ser/Thr protein kinase |
| Glyma14g05071 | PF07714 | LRR Receptor-Like Protein Kinase | |
| Glyma14g05120 | PF01603 | Serine/THREONINE PROTEIN PHOSPHATASE 2A (PP2A) REGULATORY SUBUNIT B | Serine/Threonine Protein Phosphatase 2A, Regulatory Subunit |
| Glyma14g05240 | PF00069 PF00560 PF08263 | LRR Receptor-Like Protein Kinase | Ser/Thr protein kinase |
| Glyma15g13290 | PF00931 | LRR -Containing Protein | Apoptotic Atpase |
| Glyma15g13840 | PF07714 PF00560 PF08263 | LRR Receptor-Like Protein Kinase | Ser/Thr protein kinase |
| Glyma15g14111 | PF00069 | LRR Receptor-Like Protein Kinase | |
| Glyma15g14121 | PF00069 | LRR Receptor-Like Protein Kinase | |
| Glyma18g02580.1 | PF01490 | Amino acid transporter protein | Amino acid transporter protein |
| Glyma18g02590.1 |  |  | Soluble NSF attachment protein (SNAP) |
| Glyma18g02610.1 | PF07107 | Wound-inducible protein |  |
| Glyma18g02681 | PF00069 PF00560 PF08263 | LRR Receptor-Like Protein Kinase | Ser/Thr protein kinase |
| Glyma18g02850 | PF07714 PF08263 | LRR Receptor-Like Protein Kinase | Ser/Thr protein kinase |
| Glyma18g03053 | PF07714 | LRR Receptor-Like Protein Kinase | Ser/Thr protein kinase |
| Glyma18g03066 | PF00069 | LRR Receptor-Like Protein Kinase | Ser/Thr protein kinase |
| Glyma18g03420 |  | F-Box/LRR Protein | LRR Proteins, Some Proteins Contain F-Box |
| Glyma18g03930 | PF00481 | Protein Phosphatase 2c | Serine/Threonine Protein Phosphatase |
| Glyma18g04090 | PF00069 PF00139 | LRR Receptor-Like Protein Kinase | Ser/Thr protein kinase |
| Glyma18g04340 | PF00069 | LRR Receptor-Like Protein Kinase | Ser/Thr protein kinase |
| Glyma18g04420 | PF00010 | Basic Helix-Loop-Helix (Bhlh) Family Protein Basic Helix-Loop-Helix/Leucine Zipper Transcription Factor | |
| Glyma18g04440 | PF00069 | LRR Receptor-Like Protein Kinase | Ser/Thr protein kinase |
| Glyma18g04780 | PF00560 PF00069 PF08263 | LRR Receptor-Like Protein Kinase | Ser/Thr protein kinase |
| Glyma18g04910 | PF09202 PF01163 | Serine/THREONINE-PROTEIN KINASE RIO Serine/THREONINE-PROTEIN KINASE RIO2 (RIO KINASE 2) | Ser/Thr protein kinase |
| Glyma18g04930 | PF00069 PF00139 | LRR Receptor-Like Protein Kinase | Ser/Thr protein kinase |
| Glyma18g05260 | PF01657 PF07714 | LRR Receptor-Like Protein Kinase | Ser/Thr protein kinase |
| Glyma18g05275 | PF01657 PF07714 | LRR Receptor-Like Protein Kinase | Ser/Thr protein kinase |
| Glyma18g05291 | PF01657 PF00069 | LRR Receptor-Like Protein Kinase | Ser/Thr protein kinase/Tgf-Beta Stimulated Factor |
| Glyma18g05305 | PF01657 PF00069 | LRR Receptor-Like Protein Kinase | Ser/Thr protein kinase |
| Glyma18g05710 | PF07714 PF00560 PF08263 | LRR Receptor-Like Protein Kinase | Ser/Thr protein kinase |
| Glyma18g05740 | PF00069 PF08263 PF00560 | LRR Receptor-Like Protein Kinase | Ser/Thr protein kinase |
| Glyma18g06076 | PF00069 | Ser/Thr protein kinase | Ser/Thr protein kinase |
| Glyma18g06130 | PF03822 PF00069 | Serine/THREONINE-PROTEIN KINASE PTK1,2/STK1,2 Serine/THREONINE KINASE | Ser/Thr protein kinase |
| Glyma18g06180 | PF00069 PF03822 | Serine/THREONINE KINASE PTK1,2/STK1,2 | Ser/Thr protein kinase |
| Glyma18g06300 | PF00069 | O-Sialoglycoprotein Endopeptidase | Ser/Thr protein kinase |
| Glyma18g06340.1 | PF07107 | Wound-inducible protein |  |
| Glyma18g06350.1 | PF07107 | Wound-inducible protein |  |
| Glyma18g06480 | PF07228 | Protein Phosphatase 2c | Serine/Threonine Protein Phosphatase |
| Glyma18g06630 | PF00240 PF00560 | LRR -Containing Protein | Ubiquitin-Like Protein |
| Glyma18g06810 | PF00481 | Protein Phosphatase 2c | Serine/Threonine Protein Phosphatase |
| Glyma18g07140 | PF07714 | LRR Receptor-Like Protein Kinase | Ser/Thr protein kinase |
| Glyma19g30830 | PF00450 | Serine CARBOXYPEPTIDASE II | Serine Carboxypeptidases (Lysosomal Cathepsin A) |
| Glyma19g30850 | PF00450 | Serine CARBOXYPEPTIDASE II | Serine Carboxypeptidases (Lysosomal Cathepsin A) |
| Glyma19g31271 | PF00931 | LRR -Containing Protein | |
| Glyma19g31528 | PF00931 | LRR -Containing Protein | Apoptotic Atpase |
| Glyma19g31536 | PF00931 | LRR -Containing Protein | Apoptotic Atpase |
| Glyma19g31544 | PF00931 | LRR -Containing Protein | Apoptotic Atpase |
| Glyma19g31651 | PF00931 | LRR -Containing Protein | Apoptotic Atpase |
| Glyma19g31662 | PF00931 | LRR -Containing Protein | Apoptotic Atpase |
| Glyma19g31674 | PF00560 PF00931 | LRR -Containing Protein | Apoptotic Atpase |
| Glyma19g31686 | PF00931 | LRR -Containing Protein | Apoptotic Atpase |
| Glyma19g31698 | PF00931 | LRR -Containing Protein | Apoptotic Atpase |
| Glyma19g31843 | PF00931 | LRR -Containing Protein | Apoptotic Atpase |
| Glyma19g31856 | PF00931 | LRR -Containing Protein | |
| Glyma19g31881 | PF00931 | LRR -Containing Protein | Apoptotic Atpase |
| Glyma19g32080 | PF00931 PF00560 | LRR -Containing Protein | Apoptotic Atpase |
| Glyma19g32085 | PF00560 PF00931 | LRR -Containing Protein | |
| Glyma19g32090 | PF00560 PF00931 | LRR -Containing Protein | Apoptotic Atpase |
| Glyma19g32110 | PF00931 PF00560 | LRR -Containing Protein | Apoptotic Atpase |
| Glyma19g32150 | PF00560 PF00931 | LRR -Containing Protein | Apoptotic Atpase |
| Glyma19g32200 | PF00069 PF00560 | LRR Receptor-Like Protein Kinase | Ser/Thr protein kinase |
| Glyma19g32470 | PF00069 | Ser/Thr protein kinase | Ser/Thr protein kinase |
| Glyma19g32510 | PF00069 PF00560 PF08263 | LRR Receptor-Like Protein Kinase | Ser/Thr protein kinase |
| Glyma19g32590 | PF00560 PF00069 PF08263 | LRR Receptor-Like Protein Kinase | Ser/Thr protein kinase |
| Glyma19g32700 | PF00560 PF08263 | LRR Receptor-Like Protein Kinase | |
| Glyma19g33180 | PF07714 | LRR Receptor-Like Protein Kinase | Ser/Thr protein kinase |
| Glyma19g33440 | PF07714 | LRR Receptor-Like Protein Kinase | Ser/Thr protein kinase |
| Glyma19g33451 | PF00069 | LRR Receptor-Like Protein Kinase | Ser/Thr protein kinase |
| Glyma19g33460 | PF00069 | LRR Receptor-Like Protein Kinase | Ser/Thr protein kinase |
| Glyma19g33983 |  | F-Box/LRR Protein-Related, Arath F-Box/LRR Protein | |
| Glyma19g34400 | PF00646 | F-Box/LRR Protein Subfamily | F-Box Protein Containing LRR |
| Glyma19g34920 | PF00433 PF00069 | Serine/THREONINE-PROTEIN KINASE 38 | NDR & Related Serine/Threonine Kinases |
| Glyma19g34930 | PF00069 | Casein Kinase Casein Kinase-Related | Casein Kinase (Serine/Threonine/Tyrosine Protein Kinase) |
| Glyma19g35055 | PF00560 PF08263 PF00069 | LRR Receptor-Like Protein Kinase | Ser/Thr protein kinase |
| Glyma19g35070 | PF00069 PF00560 PF08263 | LRR Receptor-Like Protein Kinase | Ser/Thr protein kinase |
| Glyma19g35190 | PF00560 PF08263 PF00069 | LRR Receptor-Like Protein Kinase | Ser/Thr protein kinase |
| Glyma19g35390 | PF07714 | LRR Receptor-Like Protein Kinase | Ser/Thr protein kinase |
| Glyma19g36090 | PF00069 | LRR Receptor-Like Protein Kinase | Ser/Thr protein kinase |
| Glyma19g36211 | PF00560 PF07714 | LRR Receptor-Like Protein Kinase | Ser/Thr protein kinase |
| Glyma19g36520 | PF00069 | LRR Receptor-Like Protein Kinase | Ser/Thr protein kinase |
| Glyma19g36700 | PF00069 | LRR Receptor-Like Protein Kinase | Ser/Thr protein kinase |
| Glyma19g36950 | PF01603 | Serine/THREONINE PROTEIN PHOSPHATASE 2A (PP2A) REGULATORY SUBUNIT B | Serine/Threonine Protein Phosphatase 2A, Regulatory Subunit |
| Glyma19g37290 | PF00069 | LRR Receptor-Like Protein Kinase | Ser/Thr protein kinase |
| Glyma19g37430 | PF07714 PF08263 PF00560 | LRR Receptor-Like Protein Kinase | Ser/Thr protein kinase |
| Glyma19g38331 | PF07714 | Leucine-Rich Receptor-Like Protein Kinase LRR Receptor-Like Protein Kinase | |
| Glyma19g39010 | PF00646 PF02373 | HISTONE ARGININE DEMETHYLASE PSR Phosphatidylserine RECEPTOR | Phosphatidylserine-Specific Receptor Ptdserr, Contains Jmjc Domain |
| Glyma19g39420 |  | Grr1-Related, Arath F-Box/LRR Protein | LRR Proteins, Some Proteins Contain F-Box |
| Glyma20g23301 | PF00931 | LRR -Containing Protein | Apoptotic Atpase |
| Glyma20g23310 | PF00149 | Serine/THREONINE PROTEIN PHOSPHATASE Serine/THREONINE PROTEIN PHOSPHATASE 6 (PP6) | Serine/Threonine Specific Protein Phosphatase Involved In Cell Cycle Control, PP2A-Related |
| Glyma20g23350 | PF02897 PF00326 | Protease Family S9a Oligopeptidase | Predicted Serine Protease |
| Glyma20g23570 |  | F-Box/LRR Protein Gb Def: Similarity To Glucose Regulated Repressor Protein (At5g23340/Mkd15_20) | LRR Proteins, Some Proteins Contain F-Box |
| Glyma20g23610 |  | LRR Receptor-Like Protein Kinase | |
| Glyma20g23880 | PF00646 | Gb Def: At2g17020 (At2g17020/At2g17020) F-Box/LRR Protein | LRR Proteins, Some Proteins Contain F-Box |
