# Supplementary material for: Genetic architecture of cyst nematode resistance revealed by genome-wide association study in soybean
Source: BMC Genomics. 2015 Aug 12;16:593. doi: 10.1186/s12864-015-1811-y (PMC4533770; doi:10.1186/s12864-015-1811-y)
Supplement: Additional file 4: Table S3. — Differential expression pattern of candidate genes evaluated in Heterodera glycines infected cells captured by laser micro-dissection & root tissue. (DOCX 28 kb) [file 12864_2015_1811_MOESM4_ESM.docx]

**Table S3** Differential expression pattern of candidate genes evaluated in *Heterodera glycines* infected cells captured by laser micro-dissection & root tissue.

| Affymatrix Probeset ID | Gene_ID (Glyma) | H. glycines infected cells captured by laser microdissection* | | | H. glycines infected root tissue # | | |
| --- | --- | --- | --- | --- | --- | --- | --- |
|  |  | 2dpi / mock | 5dpi / 2dpi | 10dpi / 2dpi | 2dpi / mock | 5dpi / mock | 10dpi / mock |
| GmaAffx.39735.1.A1_at | 01g39000 | 1.696 | 0.027 | -0.531 | 0.029 | 0.198 | -0.381 |
| GmaAffx.90673.1.S1_at | 01g39000 | 0.364 | 0.193 | -0.223 | -0.014 | 0.147 | -0.052 |
| Gma.2101.1.S1_at | 11g06250 01g39020 | -0.953 | 0.404 | -0.747 | -0.041 | -0.31 | -0.383 |
| Gma.1097.1.S1_at | 11g05830 11g05840 01g39420 | -0.553 | -0.003 | 0.069 | -0.081 | -0.269 | -0.244 |
| GmaAffx.60546.1.S1_at | 01g39470 11g05780 | -0.198 | 0.26 | 0.108 | -0.232 | -0.309 | -0.355 |
| GmaAffx.42513.1.A1_at | 04g09080 04g09090 | 0.339 | 0.609 | -0.041 | -0.055 | -0.441 | -0.311 |
| Gma.5547.1.A1_a_at | 06g09340 04g09210 | -0.014 | -1.234 | -1.151 | 0.019 | 0.228 | -0.169 |
| GmaAffx.57766.1.S1_at | 06g09340 04g09210 | -0.07 | 0.102 | 0.103 | 0.059 | 0.183 | -0.465 |
| GmaAffx.92882.1.S1_at | 04g09380 | -0.189 | -0.007 | 0.057 | -0.041 | -0.014 | 0.13 |
| GmaAffx.17951.2.S1_at | 04g09610 | -0.021 | 0.027 | 0.138 | -0.014 | 0.01 | -0.23 |
| GmaAffx.17951.1.S1_at | 04g09610 | -0.175 | 0.096 | -0.352 | 0.038 | -0.104 | -0.243 |
| Gma.869.1.S1_at | 07g31200 13g25280 | -1.189 | 2.02 | 2.58 | -0.276 | -0.523 | -0.013 |
| GmaAffx.91063.1.S1_s_at | 07g31200 13g25280 | -3.287 | 3.721 | 3.859 | -0.252 | -0.432 | -0.08 |
| GmaAffx.49400.1.S1_at | 07g31970 | 0.988 | 0.005 | -0.263 | 0.2 | 0.12 | -0.304 |
| GmaAffx.88113.1.S1_at | 07g38410 17g02350 | -0.036 | -0.026 | -0.098 | 0.078 | -0.229 | -0.068 |
| Gma.12991.1.A1_at | 07g38410 | -0.096 | 0.334 | 0.02 | -0.069 | -0.247 | -0.429 |
| Gma.10557.1.A1_at | 08g10640 | 0.09 | 0.211 | -0.238 | -0.07 | -0.424 | -0.423 |
| GmaAffx.57252.1.S1_at | 08g10680 05g27700 | -0.164 | 0.269 | 0.374 | -0.102 | -0.505 | -0.276 |
| Gma.7066.1.S1_at | 08g10680 | 0.067 | 0.533 | 0.864 | -0.062 | -0.285 | -0.274 |
| Gma.9607.1.S1_at | 05g28350 08g11350 | 0.258 | 0.19 | -0.53 | -0.067 | -0.25 | -0.097 |
| Gma.4893.1.S1_at | 08g11370 | 2.87 | 0.405 | 0.106 | 0.053 | 0.301 | 0.151 |
| Gma.16930.1.S1_at | 08g11490 05g28490 | 1.59 | -0.533 | -0.138 | -0.023 | 0.16 | -0.067 |
| Gma.13465.1.A1_at | 08g11590 | -0.33 | 1.199 | -0.338 | -0.009 | -0.352 | -0.285 |
| GmaAffx.79131.1.A1_at | 08g11590 | -0.079 | -0.056 | -0.141 | -0.137 | 0.051 | -0.103 |
| GmaAffx.79131.1.S1_at | 08g11590 05g28570 | 0.16 | 0.122 | -0.004 | -0.04 | -0.376 | -0.192 |
| GmaAffx.51412.1.S1_at | 08g11590 | -0.117 | 0.014 | 0.075 | -0.185 | -0.271 | 0.029 |
| GmaAffx.51412.2.S1_at | 08g11590 | -0.055 | -0.107 | -0.151 | -0.036 | -0.16 | -0.318 |
| Gma.5722.1.S1_at | 10g31630 20g35970 | -0.251 | 0.571 | 0.412 | -0.022 | -0.017 | -0.145 |
| GmaAffx.86702.1.S1_at | 10g31630 20g35970 | 0.378 | -0.281 | -1.056 | -0.055 | -0.039 | 0.091 |
| GmaAffx.81983.1.S1_at | 10g32090 | -0.077 | 0.067 | -0.151 | 0.017 | 0.141 | -0.306 |
| Gma.7646.2.S1_at | 10g32090 20g35520 | -0.206 | -0.136 | 0.079 | -0.051 | -0.174 | -0.388 |
| Gma.7646.1.A1_at | 10g32090 | 0.626 | -0.616 | -1.608 | 0.009 | -0.086 | -0.375 |
| GmaAffx.82485.1.S1_at | 20g35320 10g32280 | 0.005 | 0.051 | -0.012 | 0.019 | -0.329 | -0.137 |
| Gma.1065.1.S1_at | 20g35100 10g32490 | -0.326 | 0.372 | 0.113 | -0.047 | -0.126 | -0.27 |
| GmaAffx.22324.1.S1_at | 10g33970 | -0.01 | -0.233 | -0.191 | -0.217 | 0.065 | -0.129 |
| Gma.5127.1.S1_at | 10g35120 | 1.151 | -0.335 | -0.328 | -0.035 | -0.042 | -0.336 |
| GmaAffx.59065.1.S1_at | 10g35120 | 0.121 | 0.088 | -0.083 | 0.072 | 0.016 | 0.087 |
| GmaAffx.59065.2.S1_at | 10g35120 | 0.138 | -0.073 | 0.006 | -0.059 | 0.011 | -0.077 |
| Gma.6928.1.S1_at | 11g14240 12g06200 | -0.44 | -0.034 | -0.189 | 0.035 | -0.258 | -0.177 |
| GmaAffx.32694.1.S1_at | 11g15490 | 0.123 | -0.091 | -0.203 | -0.227 | -0.25 | -0.409 |
| Gma.10292.1.S1_at | 11g15550 12g07870 | -0.553 | 0.502 | -0.236 | 0.095 | -0.433 | -0.369 |
| GmaAffx.72746.1.S1_at | 01g15930 11g17120 | -0.032 | -0.117 | -0.269 | 0.13 | -0.455 | -0.944 |
| GmaAffx.49630.1.S1_at | 11g18090 | 0.101 | 0.196 | -0.36 | -0.023 | -0.279 | 0.34 |
| GmaAffx.88126.1.S1_at | 11g18090 12g10120 | -0.127 | 0.04 | -0.09 | -0.151 | -0.495 | 0.329 |
| Gma.4153.1.S1_at | 13g36600 12g33930 | -0.06 | -0.1 | -0.041 | 0.002 | -0.308 | -0.324 |
| GmaAffx.26181.1.A1_at | 13g36140 12g34410 | -0.122 | -0.05 | -0.86 | 0.091 | -0.049 | -0.147 |
| GmaAffx.26181.2.S1_at | 13g36140 12g34410 | -0.041 | 0.068 | -0.156 | 0.079 | -0.149 | -0.087 |
| Gma.18026.1.S1_at | 13g00370 | -0.177 | 0.594 | -0.498 | -0.009 | -0.464 | -0.597 |
| GmaAffx.3222.1.S1_at | 13g28570 | 0.419 | -0.769 | -1.183 | -0.022 | 0.226 | -0.473 |
| GmaAffx.55997.1.S1_at | 13g28570 15g10550 | 0.154 | -0.326 | -0.282 | -0.053 | 0.174 | -0.493 |
| PsAffx.psHB011xE10f_at | 13g28730 | -0.798 | 0.23 | -0.092 | 0.053 | -0.092 | -0.493 |
| GmaAffx.84678.1.S1_at | 13g29190 | 0.14 | -0.206 | -0.159 | -0.06 | -0.662 | -0.341 |
| Gma.4108.3.S1_a_at | 13g29410 | 0.158 | 0.384 | -0.198 | -0.023 | -0.257 | -0.26 |
| Gma.4108.1.S1_s_at | 13g29410 | -0.305 | 0.368 | 0.228 | -0.025 | -0.339 | -0.259 |
| Gma.4108.1.S1_a_at | 13g29410 15g09651 | -0.329 | 0.342 | 0.018 | 0.022 | -0.219 | -0.306 |
| Gma.4108.2.S1_at | 13g29410 15g09651 | -0.108 | 0.1 | 0.319 | -0.052 | -0.242 | -0.167 |
| GmaAffx.92151.1.S1_s_at | 02g44250 14g04520 | -0.005 | -0.023 | 0.012 | -0.002 | -0.073 | 0.266 |
| Gma.5773.1.S1_a_at | 02g44250 14g04520 | 0.096 | 0.075 | -0.009 | -0.009 | -0.06 | -0.188 |
| Gma.5773.1.S1_x_at | 02g44250 14g04520 | -0.009 | 0.213 | 0.037 | -0.007 | -0.138 | -0.212 |
| Gma.5773.4.S1_a_at | 02g44250 14g04520 | -0.728 | -0.212 | -0.335 | 0.091 | -0.207 | -1.025 |
| GmaAffx.92151.1.S1_at | 14g04520 | -0.124 | -0.032 | -0.04 | 0 | -0.141 | 0.35 |
| GmaAffx.51197.1.S1_at | 14g05120 | -0.517 | 0.141 | -0.281 | 0.013 | -0.116 | -0.323 |
| GmaAffx.51197.2.S1_at | 14g05120 02g43800 | 0.069 | 0.196 | 0.362 | -0.016 | -0.195 | -0.132 |
| GmaAffx.72394.1.S1_at | 14g05120 02g43800 | 0.019 | 0.094 | -0.229 | -0.073 | -0.111 | -0.34 |
| GmaAffx.84491.2.A1_at | 14g05240 | -0.93 | 0.694 | -1.159 | 0.052 | -0.213 | -0.307 |
| GmaAffx.39313.1.S1_at | 09g02881 15g13840 | 0.003 | -0.088 | -0.353 | -0.178 | -0.383 | -0.319 |
| GmaAffx.17305.1.S1_at | 18g02850 | -0.061 | 0.122 | 0.038 | -0.076 | 0.083 | -0.085 |
| GmaAffx.84500.1.S1_at | 11g35570 18g02850 | -0.024 | 0.212 | 0.052 | -0.001 | -0.055 | 0.012 |
| Gma.5761.1.S1_at | 11g35570 18g02850 | -0.117 | 0.588 | 0.185 | 0.001 | -0.133 | -0.252 |
| GmaAffx.92623.1.A1_at | 18g03420 | -1.129 | 0.651 | -0.242 | 0.023 | -0.066 | -0.08 |
| Gma.4474.1.S1_s_at | 11g34940 18g03420 | -0.385 | 0.629 | 0.238 | -0.068 | -0.189 | -0.038 |
| GmaAffx.92623.1.S1_at | 11g34940 18g03420 | -0.653 | 0.378 | 0.174 | -0.106 | -0.035 | -0.794 |
| Gma.4474.2.S1_at | 11g34940 18g03420 | -0.03 | -0.131 | -0.302 | 0.026 | -0.061 | -0.087 |
| GmaAffx.52826.1.S1_at | 18g03930 | -0.131 | -0.041 | -0.084 | 0.042 | -0.59 | -0.014 |
| GmaAffx.50980.1.S1_s_at | 18g03930 11g34410 | -0.27 | 0.524 | -0.292 | -0.042 | -0.443 | -0.37 |
| Gma.8058.1.A1_at | 18g03930 | -0.225 | 0.471 | -0.665 | 0.085 | -0.351 | -0.424 |
| GmaAffx.67906.1.S1_at | 18g04090 | -0.13 | -0.281 | -0.582 | -0.167 | -0.124 | -0.523 |
| GmaAffx.46686.1.S1_at | 18g04340 | 0.506 | -0.412 | -0.276 | -0.054 | 0.164 | -0.219 |
| Gma.5117.1.S1_at | 18g04780 | 0.377 | 0.254 | -0.678 | -0.123 | -0.21 | -0.59 |
| GmaAffx.82800.2.S1_at | 18g04910 11g33320 | 0.146 | -0.223 | -0.023 | -0.104 | -0.052 | -0.217 |
| GmaAffx.26299.1.S1_at | 11g31515 18g05710 | -0.435 | 1.286 | 0.413 | -0.016 | -0.108 | -0.241 |
| Gma.7673.1.S1_at | 18g05740 | -0.353 | 0.087 | -0.284 | -0.08 | -0.115 | -0.267 |
| GmaAffx.58898.1.S1_at | 18g05740 11g31440 | -0.015 | -0.057 | 0.137 | -0.158 | -0.219 | -0.087 |
| GmaAffx.60875.1.S1_at | 18g06180 | -0.179 | 0.141 | -0.205 | -0.012 | -0.308 | -0.006 |
| GmaAffx.11127.1.A1_at | 18g06180 | -1.039 | 0.806 | -0.498 | -0.074 | -0.586 | -0.751 |
| GmaAffx.51964.1.S1_at | 18g06180 | -0.086 | -0.078 | -0.19 | -0.116 | -0.561 | -0.389 |
| GmaAffx.52714.1.S1_at | 18g06300 | 0.143 | 0.008 | 0.116 | -0.022 | 0.059 | -0.105 |
| Gma.15770.1.A1_at | 18g06300 | 0.039 | -0.029 | -0.102 | -0.031 | 0.018 | -0.259 |
| Gma.2568.1.S1_at | 18g07140 | 0.297 | -0.002 | 0.075 | 0.496 | 0.344 | 0.148 |
| Gma.7489.1.A1_at | 19g30850 | -0.635 | 0.277 | 0.065 | 0.322 | 0.712 | 0.288 |
| GmaAffx.34293.1.S1_at | 19g32150 | -0.025 | 0.389 | -0.448 | 0.046 | -0.108 | -0.32 |
| GmaAffx.63850.1.S1_at | 03g29380 19g32200 | -0.132 | 0.099 | 0.037 | -0.029 | -0.294 | -0.257 |
| Gma.15656.1.S1_s_at | 03g29640 19g32470 | -0.093 | 0.686 | 0.703 | -0.096 | -0.354 | -0.421 |
| GmaAffx.92774.1.S1_at | 19g32700 | 3.425 | -0.698 | -1.855 | 0.619 | 1.895 | -0.828 |
| GmaAffx.47891.1.S1_s_at | 19g32700 | 2.962 | -0.249 | -1.447 | 0.608 | 1.683 | -0.666 |
| Gma.9825.1.S1_at | 19g33440 | -0.606 | 0.094 | 0.049 | -0.071 | -0.148 | -0.883 |
| GmaAffx.4924.1.S1_at | 03g31560 19g34400 | 0.125 | -0.211 | -0.063 | -0.107 | -0.16 | -0.152 |
| GmaAffx.45359.2.S1_at | 19g34930 | -0.36 | -0.105 | 0.223 | -0.014 | -0.054 | 0.185 |
| GmaAffx.93282.1.S1_at | 19g34930 | -0.06 | 0.064 | -0.196 | 0.049 | -0.198 | -0.258 |
| GmaAffx.45359.1.S1_at | 19g34930 | -0.416 | 0.119 | -0.545 | 0.004 | -0.101 | 0.004 |
| GmaAffx.31697.1.S1_at | 19g35390 03g32640 | -0.029 | 0.038 | -0.116 | -0.029 | -0.038 | -0.025 |
| GmaAffx.68404.1.S1_at | 19g39420 | -0.025 | 0.195 | -0.027 | 0.05 | -0.071 | 0.139 |
| GmaAffx.36982.1.S1_at | 19g39420 | -0.368 | -0.019 | -0.118 | 0.015 | -0.038 | 0.245 |
| Gma.1557.1.S1_a_at | 03g36770 19g39420 | -0.414 | 0.224 | 0.019 | 0.017 | -0.113 | -0.033 |
| GmaAffx.90260.1.S1_s_at | 10g43464 10g43480 20g23310 0048s00290 | 0.402 | -0.152 | 0.255 | -0.049 | -0.02 | -0.109 |
| Gma.5883.1.S1_a_at | 10g43464 10g43480 20g23310 | 0.128 | 0.069 | -0.157 | 0.051 | -0.046 | -0.333 |
| Gma.5883.1.S1_at | 20g23310 | 0.16 | -0.007 | -0.275 | 0.032 | 0.018 | -0.268 |
| GmaAffx.90260.1.S1_at | 10g43464 20g23310 | 0.607 | 0.027 | 0.396 | -0.007 | 0.011 | -0.013 |
| GmaAffx.58279.1.S1_at | 20g23350 | 0.046 | -0.006 | -0.17 | -0.039 | 0.044 | 0.038 |
| GmaAffx.71561.1.S1_at | 20g23350 | -0.074 | 0.022 | -0.164 | -0.051 | 0.153 | -0.141 |
| Gma.17896.1.S1_at | 20g23350 | 0.819 | -0.459 | -0.287 | 0.018 | 0.138 | -0.357 |
| GmaAffx.93465.1.S1_at | 20g23570 | -0.227 | 0.414 | 0.232 | 0.081 | -0.147 | -0.634 |
| GmaAffx.93465.1.A1_s_at | 20g23570 | -0.131 | 0.395 | 0.247 | -0.039 | -0.273 | -0.222 |
| GmaAffx.38743.1.S1_at | 20g23570 | -0.053 | 0.008 | 0.028 | -0.016 | -0.294 | -0.005 |
