# Supplementary material for: Genetic architecture of cyst nematode resistance revealed by genome-wide association study in soybean
Source: BMC Genomics. 2015 Aug 12;16:593. doi: 10.1186/s12864-015-1811-y (PMC4533770; doi:10.1186/s12864-015-1811-y)
Supplement: Additional file 5: Figure S2. — Significantly associated SNPs with the flower color in soybean identified by genome-wide association study (GWAS). (DOCX 635 kb) [file 12864_2015_1811_MOESM5_ESM.docx]

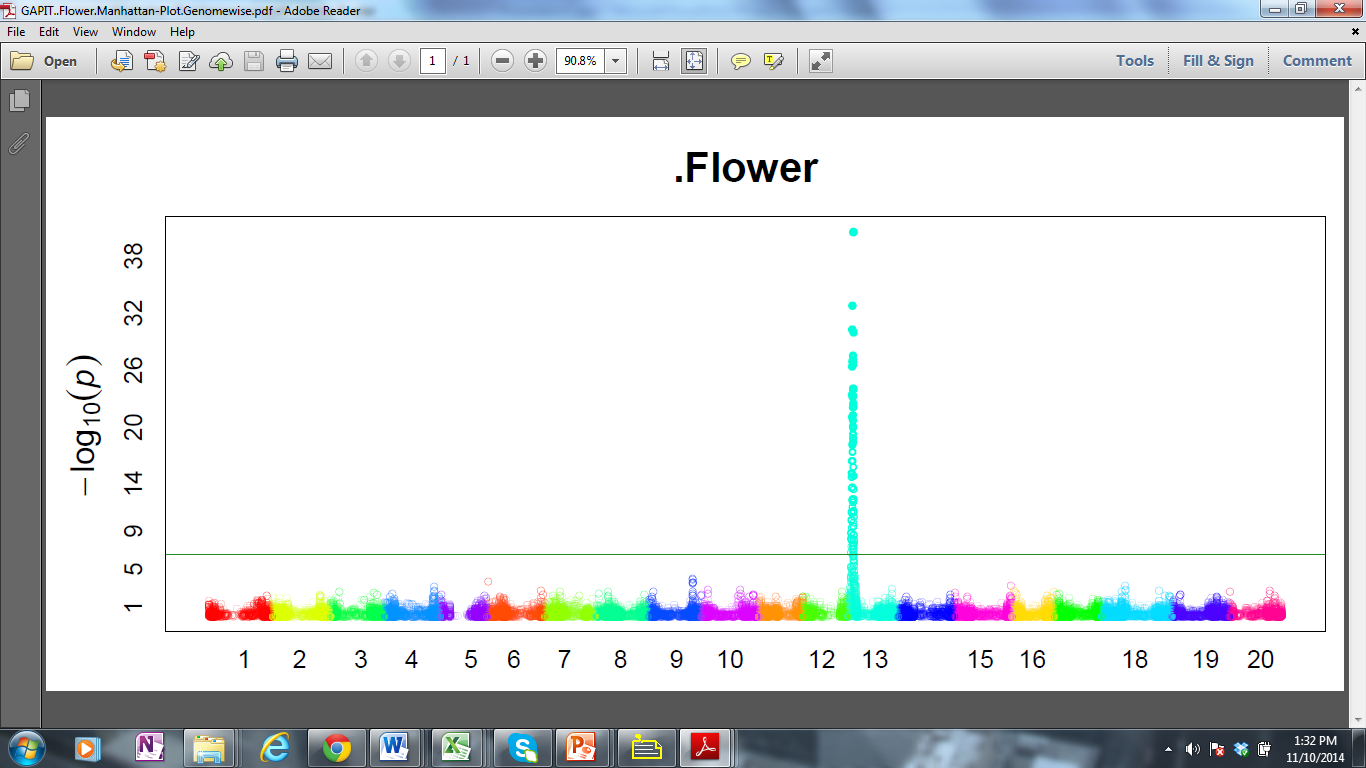


Flower color

Chromosome

**Figure S2** Significantly associated single nucleotide polymorphism (SNP) with the flower color in soybean identified by genome-wide association study (GWAS).
