# Supplementary material for: Genetic architecture of cyst nematode resistance revealed by genome-wide association study in soybean
Source: BMC Genomics. 2015 Aug 12;16:593. doi: 10.1186/s12864-015-1811-y (PMC4533770; doi:10.1186/s12864-015-1811-y)
Supplement: Additional file 6: Table S4. — A comparison of genome-wide association study (GWAS) loci identified in the present study and quantitative trait loci (QTL) previously reported for resistance to soybean cyst nematode (SCN) from different sources of resistance showing the consistency of genetic locations of GWAS and QTL analysis. (DOCX 13 kb) [file 12864_2015_1811_MOESM6_ESM.docx]

**Table S4** A comparison of genome-wide association study (GWAS) loci identified in the present study and quantitative trait loci (QTL) previously reported for resistance to soybean cyst nematode (SCN) from different sources of resistance showing the consistency of genetic locations of GWAS and QTL analysis.

| **Chromo-**  **some** | **Linkage Group** | **Source of resistance** | **Tested Race (†) (HG Types)** | **Publication** |
| --- | --- | --- | --- | --- |
| 1 | D1a | PI 89772, PI 438489B | 1, 2, 3, 5, 14 | Yue et al. (2001), Vuong et al. (2011) |
| 4 | C1 | PI 437654, PI 438489B, PI 467312 | 1, 2, 3, 5, 14 | Webb (2003), Vuong et al. (2011), Wu et al. (2009) Yue et al. (2001) |
| 7 | M | PI 437654 | 1, 3, 5, 14 | Webb (1995), Wu et al. (2009) |
| 8 | A2 | Peking, PI 88788, PI 437654,  PI 209332 | 1, 3 | Mekemse et al. (2001), Concibido et al. (1994), Wu et al. (2009), Webb (2003) |
| 10 | O | PI 567516C | 1, 2, 3, 5, 14, LY1 | Vuong et al. (2010) |
| 11 | B1 | Peking, PI 437654, PI 89772,  PI 438489B, | 1, 2, 3, 5 | Vierling et al. (1996), Yue et al. (2001), Webb, (2003), Wu et al. (2009) |
| 12 | H | Peking | 1, 3 | Qui et al. (1999) |
| 13 | F | Peking, PI 437654, PI 88788,  PI 90763 | 1, 3, 5 | Mahalingam and Skorupska (1995), Vierling et al. (1996), Heer et al. (1998) |
| 14 | B2 | Peking, PI 438489B | 1, 3 | Qui et al. (1999), Yue et al. (2001) |
| 15 | E | PI 438489B, PI 468916, PI 89772 | 2, 3, 14 | Yue et al. (2001), Wang et al. (2001) |
| 18 | G | Peking, PI 209332, PI 437654,  PI 88788, PI 438489B, PI 90763, PI 209332, PI 468916, PI 89772 | 1, 2, 3, 5, 6, 14 | Concibido et al. (1994, 1997, 1997, 2004), Mekseme et al. (2001), Vuong et al. (2011),Webb (2003), Wang et al. (2001), Wu et al. (2009), Yue et al. 2001) |
| 19 | L | PI 209332, PI 90763 | 1, 2, 3, 5 | Concibido et al. (1997), Guo et al. (2005) |
| 20 | I | Peking, PI 437654, PI 437655 | 2, 3, 5, 14, LY2 | Qui et al. (1999), Jiao et al. (2014) |

(†): Race classification system was commonly utilized to identify responses to specific nematode populations in many previous genetic analysis.
