# Supplementary material for: Genetic architecture of cyst nematode resistance revealed by genome-wide association study in soybean
Source: BMC Genomics. 2015 Aug 12;16:593. doi: 10.1186/s12864-015-1811-y (PMC4533770; doi:10.1186/s12864-015-1811-y)
Supplement: Additional file 7: Table S5. — Five hundred fifty three soybean germplasm accessions with morphological characteristics, agronomic traits, and code definitions were utilized in genome-wide association study. (DOCX 61 kb) [file 12864_2015_1811_MOESM7_ESM.docx]

**Table S5** Five hundred fifty three soybean germplasm accessions with morphological characteristics, agronomic traits, and code definitions were utilized in genome-wide association study.

| **No** | **PI line** | **Stem growth habit** | **Flower color** | **Hilum color** | **Pubescence color** | **Seed coat color** | **Lodging** | **Maturity group** |
| --- | --- | --- | --- | --- | --- | --- | --- | --- |
| 1 | PI 60273 | N | P | Brbl | T | Gn | 4.0 | V |
| 2 | PI 60296 | N | W | Bf | G | Y | 4.0 | V |
| 3 | PI 79691-4 | D | W | Br | T | Br | 3.3 | III |
| 4 | PI 80461 | N | W | Bf | G | Y | 3.3 | III |
| 5 | PI 80473 | N | W | Bf | G | Y | 3.8 | IV |
| 6 | PI 80479 | N | W | Bl | T | Y | 3.0 | IV |
| 7 | PI 80488 | D | W | Br | T | Y | 3.0 | IV |
| 8 | PI 80831 | N | W | Y | G | Y | 2.5 | III |
| 9 | PI 80834-1 | N | W | Bf | G | Y | 2.3 | IV |
| 10 | PI 80847-2 | N | P | Bl | T | Y | 2.7 | IV |
| 11 | PI 81029-1 | D | W | Bl | T | Gn | 2.9 | IV |
| 12 | PI 81034-1 | N | P | Y | G | Y | 3.9 | IV |
| 13 | PI 81042-1 | N | P | Ib | G | Gn | 2.8 | IV |
| 14 | PI 81044-2 | N | W | Bf | G | Gn | 3.0 | III |
| 15 | PI 81667 | D | P | Br | T | Br | 2.9 | III |
| 16 | PI 81766 | N | Dp | Bl | T | Bl | 4.5 | III |
| 17 | PI 83925 | N | P | Lbf | G | Y | 4.0 | IV |
| 18 | PI 84594 | N | P | Gn | G | Gn | 4.0 | IV |
| 19 | PI 84611 | N | P | Bl | Lt | Bl | 3.4 | III |
| 20 | PI 84632S | N | W | Bf | G | Gn | 4.0 | V |
| 21 | PI 84646-2 | N | P | Bf | - | Y | 3.8 | IV |
| 22 | PI 84669 | N | W | Bf | G | Y | 4.0 | V |
| 23 | PI 84679 | N | P | Bf | G | Y | 3.2 | IV |
| 24 | PI 84751 | D | W | Bl | T | Bl | 2.7 | IV |
| 25 | PI 84973 | N | W | Bf | - | Y | 3.2 | III |
| 26 | PI 85009-1 | N | W | Br | T | Gn | 4.3 | III |
| 27 | PI 85089 | N | W | Bf | G | Y | 4.0 | V |
| 28 | PI 85355 | D | P | Ib | G | Y | 3.5 | IV |
| 29 | PI 85356 | N | W | Br | T | Y | 3.1 | III |
| 30 | PI 86045S | N | P | Brbl | T | Gn | 5.0 | V |
| 31 | PI 86084 | N | P | G | G | Y | 5.0 | V |
| 32 | PI 86113S | N | P | Bf | G | Y | 5.0 | V |
| 33 | PI 86134-4 | N | W | Br | T | Y | 3.8 | IV |
| 34 | PI 86145 | D | W | Br | Ng | Br | 2.7 | III |
| 35 | PI 86150 | D | P | Bf | G | Y | 4.0 | IV |
| 36 | PI 86452 | D | P | Bf | - | Y | 2.5 | III |
| 37 | PI 86465 | N | W | Bl | T | Y | 4.5 | V |
| 38 | PI 86982 | N | P | Bf | G | Y | 5.0 | V |
| 39 | PI 87037 | N | P | Br | T | Y | 5.0 | V |
| 40 | PI 87076 | N | P | Bf | G | Y | 5.0 | V |
| 41 | PI 87629 | N | P | Br | T | Br | 4.0 | IV |
| 42 | PI 87631-1 | N | P | Bl | T | Lgn | 4.5 | III |
| 43 | PI 87632 | N | P | Br | T | Y | 4.8 | IV |
| 44 | PI 87634 | N | P | Br | T | Y | 3.5 | III |
| 45 | PI 88292 | N | W | Bl | T | Bl | 4.3 | III |
| 46 | PI 88305 | S | W | Bf | G | Y | 2.0 | III |
| 47 | PI 88306 | D | W | Lbf | G | Y | 3.4 | III |
| 48 | PI 88444 | D | W | Bf | G | Y | 3.6 | IV |
| 49 | PI 88788 | N | W | Bl | T | Bl | 4.2 | III |
| 50 | PI 88820 | N | W | Bl | T | Y | 4.5 | V |
| 51 | PI 89061-3 | N | W | Br | Lt | Y | 3.6 | IV |
| 52 | PI 89128 | N | P | Bl | T | Y | 3.6 | IV |
| 53 | PI 89130 | N | W | Y | G | Y | 3.1 | III |
| 54 | PI 89134 | N | P | Tn | T | Y | 3.3 | III |
| 55 | PI 89152 | N | P | Ib | G | Y | 3.2 | III |
| 56 | PI 89772 | N | P | Bl | T | Bl | 4.0 | IV |
| 57 | PI 89773 | D | W | Bf | G | Gn | 3.5 | III |
| 58 | PI 90221 | N | P | Bf | G | Y | 3.8 | IV |
| 59 | PI 90245 | N | W | Y | G | Y | 3.7 | IV |
| 60 | PI 90369 | D | W | Bf | G | Y | 3.0 | IV |
| 61 | PI 90401 | D | W | Bf | G | Y | 2.4 | IV |
| 62 | PI 90479P | D | W | Y | G | Y | 3.0 | IV |
| 63 | PI 90563 | D | P | Y | G | Y | 4.0 | IV |
| 64 | PI 90763 | N | P | Bl | Lt | Bl | 3.4 | IV |
| 65 | PI 91082 | N | W | Y | G | Y | 3.0 | IV |
| 66 | PI 91120-3 | N | P | Bl | T | Y | 2.0 | III |
| 67 | PI 91162 | D | P | Br | T | Y | 4.5 | III |
| 68 | PI 91341 | D | P | Bl | T | Gn | 4.0 | III |
| 69 | PI 91349 | N | P | Br | T | Bl | 4.8 | III |
| 70 | PI 91679 | N | W | Lbf | G | Y | 3.6 | IV |
| 71 | PI 91725 | N | W | Y | G | Y | 4.0 | V |
| 72 | PI 92636 | D | P | Y | G | Y | 3.3 | IV |
| 73 | PI 96199 | D | W | Bf | G | Y | 4.0 | III |
| 74 | PI 96322 | N | W | Bl | T | Bl | 2.4 | III |
| 75 | PI 96333 | N | P | Ib | G | Gn | 3.4 | IV |
| 76 | PI 96786-1 | N | W | Lbf | G | Y | 3.4 | III |
| 77 | PI 96808 | D | W | Y | G | Y | 4.0 | IV |
| 78 | PI 96984 | N | P | Bl | T | Y | 3.2 | IV |
| 79 | PI 123577B | N | W | Bf | G | Y | 4.3 | IV |
| 80 | PI 123590 | N | W | Y | G | Y | 5.0 | V |
| 81 | PI 157405 | D | P | G | G | Lgn | 4.0 | IV |
| 82 | PI 157406 | N | W | Bf | G | Y | 4.5 | V |
| 83 | PI 157413 | N | W | Bf | G | Y | 4.0 | V |
| 84 | PI 157468 | D | W | Gn | T | Gn | 4.2 | IV |
| 85 | PI 157485 | N | P | Bf | G | Y | 3.7 | IV |
| 86 | PI 167240 | N | W | Y | G | Y | 2.8 | III |
| 87 | PI 171429 | D | W | Blbr | T | Bl | 4.8 | IV |
| 88 | PI 171449 | D | P | Bf | - | Y | 3.8 | III |
| 89 | PI 172902 | D | W | Lgn | T | Lgn | 2.5 | V |
| 90 | PI 173994 | D | W | Y | G | Y | 4.5 | III |
| 91 | PI 179826 | N | W | Br | Ng | Br | 3.3 | IV |
| 92 | PI 196149 | D | W | Bl | T | Bl | 2.4 | III |
| 93 | PI 200460 | D | P | Lbr | T | Y | 3.8 | IV |
| 94 | PI 200470 | D | P | Y | - | Y | 4.3 | IV |
| 95 | PI 200478 | D | W | Br | - | Y | 3.0 | III |
| 96 | PI 200501 | D | W | Br | T | Y | 4.3 | IV |
| 97 | PI 200548 | D | W | Bf | G | Gn | 3.3 | III |
| 98 | PI 205086 | D | P | Bf | - | Y | 3.0 | III |
| 99 | PI 205087 | D | P | Br | - | Y | 1.5 | III |
| 100 | PI 205088 | D | W | Br | T | Y | 4.0 | IV |
| 101 | PI 209332 | N | P | Bl | T | Bl | 4.0 | IV |
| 102 | PI 210179 | D | P | Br | T | Y | 4.0 | V |
| 103 | PI 219782 | D | P | Bf | G | Y | 3.7 | IV |
| 104 | PI 224271 | D | B | Br | T | Y | 4.4 | IV |
| 105 | PI 226591 | D | B | Y | G | Y | 3.8 | IV |
| 106 | PI 227212 | D | P | Br | - | Y | 3.8 | III |
| 107 | PI 229327 | D | P | Br | T | Y | 3.7 | IV |
| 108 | PI 229336 | D | P | Bf | - | Y | 2.8 | III |
| 109 | PI 229342 | D | W | Br | T | Y | 3.6 | IV |
| 110 | PI 229343 | D | W | Br | T | Y | 3.0 | IV |
| 111 | PI 229352 | D | P | Bf | G | Y | 3.4 | IV |
| 112 | PI 229356 | D | W | Lbf | G | Y | 3.9 | IV |
| 113 | PI 229361 | D | P | Br | T | Y | 3.9 | IV |
| 114 | PI 229362 | D | P | Bf | G | Y | 3.8 | IV |
| 115 | PI 235347 | D | W | Y | G | Y | 4.0 | IV |
| 116 | PI 238928 | D | P | Br | T | Y | 1.5 | IV |
| 117 | PI 238929 | D | W | Br | T | Y | 1.0 | V |
| 118 | PI 243514 | N | W | Br | T | Y | 2.8 | IV |
| 119 | PI 243515 | D | P | Bl | T | Bl | 4.0 | IV |
| 120 | PI 243522 | D | P | Br | T | Y | 2.5 | IV |
| 121 | PI 243524 | D | W | Br | T | Gn | 3.2 | IV |
| 122 | PI 243527 | D | W | Bl | T | Bl | 3.5 | IV |
| 123 | PI 243533 | N | P | Gn | T | Gn | 4.4 | IV |
| 124 | PI 243540 | D | P | Br | T | Y | 4.5 | IV |
| 125 | PI 243541 | D | W | Br | T | Y | 3.8 | IV |
| 126 | PI 243544 | D | B | Bf | G | Y | 3.8 | IV |
| 127 | PI 243545 | D | W | Rbr | T | Rbr | 3.2 | IV |
| 128 | PI 243546 | D | P | Br | T | Br | 4.7 | IV |
| 129 | PI 243548 | D | P | Gn | G | Gn | 3.4 | IV |
| 130 | PI 246369 | D | P | Br | T | Y | 4.5 | IV |
| 131 | PI 248515 | D | W | Y | G | Y | 3.2 | IV |
| 132 | PI 253651C | N | P | Bl | Lt | Bl | 3.8 | III |
| 133 | PI 253656A | N | P | Bf | G | Gn | 4.8 | IV |
| 134 | PI 253666A | N | W | Bf | G | Y | 4.8 | IV |
| 135 | PI 261466 | D | W | Br | T | Gn | 4.3 | III |
| 136 | PI 262181 | D | P | Lbf | G | Y | 3.8 | IV |
| 137 | PI 274420 | D | P | Y | G | Y | 1.5 | IV |
| 138 | PI 303652 | N | P | Bl | T | Bl | 4.5 | V |
| 139 | PI 323555 | N | W | Blbr | Lt | Y | 3.3 | IV |
| 140 | PI 339734 | N | P | Bl | T | Bl | 4.5 | IV |
| 141 | PI 339736 | N | P | Br | T | Gnbr | 4.3 | IV |
| 142 | PI 339868B | D | W | Bl | T | Bl | 2.3 | IV |
| 143 | PI 339980 | D | P | Bl | T | Bl | 1.5 | V |
| 144 | PI 339984 | D | P | Lbf | G | Lgn | 2.0 | IV |
| 145 | PI 339986 | D | P | Y | G | Y | 1.0 | V |
| 146 | PI 340023 | D | P | Y | G | Y | 2.0 | V |
| 147 | PI 340042 | N | P | Bl | T | Y | 3.8 | IV |
| 148 | PI 346308 | D | P | Tn | T | Y | 2.5 | IV |
| 149 | PI 346309 | S | P | Tn | T | Y | 5.0 | V |
| 150 | PI 361101 | N | W | Y | G | Y | 2.6 | III |
| 151 | PI 361103 | D | P | Y | G | Y | 2.5 | IV |
| 152 | PI 378682A | N | P | Bl | T | Bl | 5.0 | IV |
| 153 | PI 379561 | D | W | Br | Lt | Br | 2.1 | III |
| 154 | PI 391597 | D | W | Lbr | T | Y | 2.5 | V |
| 155 | PI 398206 | N | P | Y | T | Y | 4.0 | IV |
| 156 | PI 398229 | D | P | Bf | G | Y | 1.8 | IV |
| 157 | PI 398305 | N | P | Gn | G | Gn | 3.5 | IV |
| 158 | PI 398447 | D | P | Bl | T | Bl | 2.0 | V |
| 159 | PI 398465 | N | P | Br | T | Y | 4.5 | V |
| 160 | PI 398491 | D | P | Y | G | Y |  | V |
| 161 | PI 398513 | D | P | Gn | G | Gn | 1.5 | V |
| 162 | PI 398525 | D | P | Gn | G | Gn | 2.0 | V |
| 163 | PI 398568 | D | P | Bl | Lt | Bl | 1.5 | V |
| 164 | PI 398589 | D | P | Br | T | Y | 3.0 | V |
| 165 | PI 398593 | D | P | Br | T | Y | 3.0 | V |
| 166 | PI 398595 | D | P | Br | T | Y | 3.0 | V |
| 167 | PI 398612 | D | P | Br | T | Y | 3.0 | V |
| 168 | PI 398649 | D | W | Rbf | G | Rbf | 1.5 | V |
| 169 | PI 398677 | D | W | Bl | T | Bl | 3.5 | V |
| 170 | PI 398705 | N | P | Bl | T | Y | 3.8 | IV |
| 171 | PI 398777 | D | P | Gn | G | Gn | 2.0 | V |
| 172 | PI 398802 | D | P | Bl | T | Bl | 2.5 | IV |
| 173 | PI 398813 | S | P | Y | G | Y | 1.5 | III |
| 174 | PI 398818 | D | P | Rbr | T | Rbr | 2.5 | V |
| 175 | PI 398819 | N | P | Br | Lt | Y | 4.5 | V |
| 176 | PI 398828 | D | P | Bl | T | Bl | 3.5 | V |
| 177 | PI 398844 | D | P | Gn | G | Gn | 2.5 | V |
| 178 | PI 398884 | D | P | Bl | T | Bl | 2.3 | IV |
| 179 | PI 398965 | N | W | Bl | T | Bl | 2.1 | IV |
| 180 | PI 398972 | D | W | Bf | G | Y | 2.0 | V |
| 181 | PI 399007 | D | P | Brbl | Ng | Gn | 3.5 | V |
| 182 | PI 399076 | N | P | Gnbr | T | Gnbr | 4.5 | V |
| 183 | PI 399100 | N | P | Br | T | Gn | 4.5 | V |
| 184 | PI 399103 | D | W | G | T | Ggn | 3.5 | V |
| 185 | PI 404166 | N | P | Bl | T | Bl | 2.5 | III |
| 186 | PI 404169B | S | P | Ib | G | Y | 3.9 | III |
| 187 | PI 404198B | N | P | Bl | T | Bl | 5.0 | IV |
| 188 | PI 407653 | N | P | Bl | Ng | Bl | 3.4 | III |
| 189 | PI 407729 | N | W | Bl | Lt | Bl | 3.3 | IV |
| 190 | PI 407746 | D | W | Bl | Lt | Bl | 2.0 | III |
| 191 | PI 407759 | S | P | Br | T | Br | 3.0 | V |
| 192 | PI 407761 | D | P | Br | Lt | Br | 3.5 | V |
| 193 | PI 407778A | N | P | Bl | T | Bl | 3.3 | IV |
| 194 | PI 407810 | N | P | Bl | T | Bl | 3.1 | III |
| 195 | PI 407833B | N | W | Br | T | Y | 4.5 | V |
| 196 | PI 407836 | D | P | Bl | T | Bl | 4.0 | V |
| 197 | PI 407842 | N | P | Bl | T | Bl | 4.5 | V |
| 198 | PI 407866 | D | P | Bl | T | Bl | 2.5 | V |
| 199 | PI 407890-1 | D | P | Gn | G | Gn | 1.5 | V |
| 200 | PI 407940 | N | P | Bf | G | Y | 4.5 | V |
| 201 | PI 407941A | D | P | Gn | G | Gn | 1.5 | V |
| 202 | PI 407957 | D | P | Bl | T | Bl | 4.0 | V |
| 203 | PI 407965 | N | P | Bf | G | Y | 4.5 | V |
| 204 | PI 407986B | N | P | Bl | T | Y | 4.5 | V |
| 205 | PI 408012 | N | P | Gnbr | T | Gnbr | 5.0 | V |
| 206 | PI 408023 | D | P | Br | T | Br | 2.0 | V |
| 207 | PI 408056 | D | W | Y | G | Y | 4.0 | V |
| 208 | PI 408063 | D | W | Bf | G | Y | 3.5 | V |
| 209 | PI 408088 | N | P | Gnbr | Lt | Gnbr | 5.0 | V |
| 210 | PI 408096 | N | W | Br | T | Gn | 5.0 | V |
| 211 | PI 408107 | N | P | Br | Lt | Br | 5.0 | V |
| 212 | PI 408113 | D | P | Bf | G | Gn | 3.0 | V |
| 213 | PI 408123 | N | P | Bl | T | Bl | 5.0 | V |
| 214 | PI 408134B | D | P | Gn | T | Gn | 1.5 | V |
| 215 | PI 408150 | N | P | Bf | G | Gn | 5.0 | V |
| 216 | PI 408167A | D | W | Bf | G | Y | 3.5 | V |
| 217 | PI 408183 | D | P | Ib | G | Gn | 3.5 | V |
| 218 | PI 408207-1 | D | W | Bf | G | Y | 2.0 | V |
| 219 | PI 408251 | N | P | Bl | T | Bl | 4.5 | V |
| 220 | PI 408304 | N | P | Br | T | Gn | 5.0 | V |
| 221 | PI 408337 | D | W | Br | T | Y | 1.5 | V |
| 222 | PI 416771 | D | P | Rbr | T | Rbr | 3.0 | V |
| 223 | PI 416799 | N | P | Br | T | Gn | 3.0 | V |
| 224 | PI 416807 | D | W | Bl | T | Y | 3.0 | V |
| 225 | PI 416821 | D | W | Rbr | T | Rbr | 2.0 | V |
| 226 | PI 416838 | D | W | Y | G | Y | 2.0 | V |
| 227 | PI 416843 | D | W | Y | T | Y | 3.0 | V |
| 228 | PI 416844 | D | P | Y | G | Y | 2.0 | V |
| 229 | PI 416850 | D | W | Br | T | Y | 2.0 | V |
| 230 | PI 416859 | N | P | Br | T | Y | 1.5 | IV |
| 231 | PI 416868A | N | W | Bl | T | Y | 3.0 | III |
| 232 | PI 416871 | D | W | Y | G | Y | 3.0 | V |
| 233 | PI 416892 | D | P | Bl | T | Bl | 2.8 | III |
| 234 | PI 416902 | N | W | Lbf | G | Y | 2.3 | III |
| 235 | PI 416908 | D | P | Lbl | T | Y | 2.0 | V |
| 236 | PI 416971 | N | W | Bf | G | Y | 3.8 | IV |
| 237 | PI 416984 | D | W | Bf | G | Y | 3.8 | IV |
| 238 | PI 417026 | D | P | Bf | G | Y | 5.0 | V |
| 239 | PI 417056 | D | P | Y | G | Y | 1.3 | IV |
| 240 | PI 417058 | D | P | Bl | T | Bl | 4.0 | V |
| 241 | PI 417065 | D | P | Bf | - | Y | 1.5 | IV |
| 242 | PI 417090 | N | P | Bl | T | Bl | 3.0 | V |
| 243 | PI 417091 | N | P | Bl | T | Bl | 4.4 | II |
| 244 | PI 417092 | N | Dp | Bl | T | Bl | 3.8 | IV |
| 245 | PI 417093 | N | P | Bl | Ng | Bl | 5.0 | V |
| 246 | PI 417135A | N | P | Bl | T | Y | 3.0 | IV |
| 247 | PI 417159 | D | P | Y | G | Y | 2.0 | V |
| 248 | PI 417167 | D | P | Br | - | Br | 1.5 | III |
| 249 | PI 417168 | D | W | Br | - | Bl | 1.3 | IV |
| 250 | PI 417198 | D | P | Br | T | Y | 2.1 | III |
| 251 | PI 417209 | D | P | Br | T | Y | 2.0 | V |
| 252 | PI 417217 | N | W | Bf | G | Y | 2.5 | IV |
| 253 | PI 417275 | N | W | Bf | G | Y | 3.5 | V |
| 254 | PI 417297 | D | P | Bl | - | Bl | 1.5 | III |
| 255 | PI 417339 | D | W | Y | G | Y | 1.2 | IV |
| 256 | PI 417373 | D | P | Y | G | Y | 3.0 | V |
| 257 | PI 417380 | D | W | Gn | T | Gn | 2.3 | IV |
| 258 | PI 417415 | D | W | Y | G | Y | 3.0 | V |
| 259 | PI 417430 | D | W | Y | G | Y | 2.5 | V |
| 260 | PI 417435 | D | W | Y | G | Y | 1.3 | IV |
| 261 | PI 417486 | D | P | Bl | T | Y | 3.0 | V |
| 262 | PI 417559 | S | W | Br | T | Y | 2.0 | III |
| 263 | PI 423743B | D | P | Bl | T | Gn | 1.0 | V |
| 264 | PI 423764 | D | W | Y | G | Y | 2.5 | V |
| 265 | PI 423787 | N | P | Br | T | Y | 3.5 | IV |
| 266 | PI 423799C | D | P | Rbr | Lt | Rbr | 2.5 | V |
| 267 | PI 423813 | N | P | Bl | T | Bl | 1.8 | IV |
| 268 | PI 423827A | N | P | Lbf | G | Y | 5.0 | IV |
| 269 | PI 423833A | N | W | Bl | T | Ggn | 4.5 | IV |
| 270 | PI 423841 | N | P | Bf | G | Y | 4.0 | IV |
| 271 | PI 423842 | S | W | Bl | T | Ggn | 4.0 | IV |
| 272 | PI 423850 | N | P | Y | G | Y | 3.5 | IV |
| 273 | PI 423897 | D | W | Y | G | Y | 2.0 | V |
| 274 | PI 423912 | D | W | Y | G | Y | 2.5 | V |
| 275 | PI 424005 | N | W | Brbl | T | Gn | 5.0 | III |
| 276 | PI 424024 | N | P | Bl | T | Bl | 3.8 | IV |
| 277 | PI 424078 | N | P | Bl | T | Bl | 2.8 | III |
| 278 | PI 424154B | N | P | Rbr | T | Rbr | 1.5 | IV |
| 279 | PI 424159B | N | W | Y | T | Y | 2.2 | IV |
| 280 | PI 424275 | N | W | Bl | T | Gn | 4.5 | IV |
| 281 | PI 424293 | D | W | Bf | G | Y | 1.8 | IV |
| 282 | PI 424294A | N | W | Bl | T | G | 3.3 | IV |
| 283 | PI 424298 | N | P | Bl | T | Bl | 4.5 | IV |
| 284 | PI 424431 | N | P | Bl | T | Bl | 3.3 | IV |
| 285 | PI 424460 | N | W | Bl | T | Ggn | 4.0 | IV |
| 286 | PI 424498 | N | W | Bl | T | Ggn | 4.5 | IV |
| 287 | PI 424513 | D | W | Y | G | Y | 2.3 | IV |
| 288 | PI 424525 | N | P | Bf | G | Gn | 3.0 | IV |
| 289 | PI 424556 | D | P | Bl | T | Bl | 1.5 | IV |
| 290 | PI 424564 | D | P | Bl | T | Gn | 1.8 | IV |
| 291 | PI 424607 | N | P | Br | T | Br | 3.5 | IV |
| 292 | PI 424608A | N | P | Bl | T | Bl | 2.8 | IV |
| 293 | PI 430619 | N | W | Br | T | Y | 2.8 | III |
| 294 | PI 437654 | N | P | Bl | T | Bl | 4.5 | III |
| 295 | PI 437655 | N | P | Bl | Lt | Bl | 4.3 | III |
| 296 | PI 437679 | S | P | Bl | T | Bl | 3.0 | IV |
| 297 | PI 437690 | S | W | Bl | Lt | Bl | 2.3 | III |
| 298 | PI 437725 | S | P | Bl | T | Bl | 2.5 | IV |
| 299 | PI 437770 | N | W | Bl | Ng | Bl | 3.0 | III |
| 300 | PI 438303 | N | P | Bl | T | Bl | 4.8 | IV |
| 301 | PI 438312 | N | P | Bl | T | Bl | 4.0 | III |
| 302 | PI 438424 | D | P | Y | G | Y | 3.5 | IV |
| 303 | PI 438425 | D | P | Y | G | Y | 2.5 | V |
| 304 | PI 438489B | N | P | Bl | T | Bl | 5.0 | IV |
| 305 | PI 438497 | D | W | Bl | T | Bl | 2.3 | III |
| 306 | PI 438503A | N | W | Bl | Ng | Bl | 3.0 | II |
| 307 | PI 442017 | D | P | Y | G | Y | 1.0 | V |
| 308 | PI 445845 | D | W | Bf | G | Y | 3.0 | III |
| 309 | PI 458020 | N | W | Bl | Lt | Ggn | 4.0 | IV |
| 310 | PI 458052 | D | P | Y | G | Y | 2.5 | III |
| 311 | PI 458053A | D | W | Bl | T | Bl | 2.0 | IV |
| 312 | PI 458060A | D | W | Bl | T | Bl | 2.0 | IV |
| 313 | PI 458061A | D | W | Y | G | Y | 1.3 | III |
| 314 | PI 458070A | D | W | Bl | T | Bl | 2.0 | IV |
| 315 | PI 458071 | D | W | Bl | T | Bl | 2.0 | IV |
| 316 | PI 458081 | D | W | Y | G | Y | 2.5 | V |
| 317 | PI 458103 | N | P | Lbf | G | Y | 4.8 | IV |
| 318 | PI 458110 | D | P | Y | G | Y | 2.3 | III |
| 319 | PI 458118 | D | W | Bf | G | Y | 2.3 | IV |
| 320 | PI 458137 | D | P | Bl | T | Bl | 3.5 | IV |
| 321 | PI 458166 | D | P | Bl | T | Bl | 1.5 | IV |
| 322 | PI 458181 | D | P | Br | T | Br | 3.8 | IV |
| 323 | PI 458184 | D | P | Bl | T | Bl | 5.0 | IV |
| 324 | PI 458224 | N | W | Bf | G | Y | 4.5 | IV |
| 325 | PI 458231 | D | P | Bf | G | Gn | 4.3 | IV |
| 326 | PI 458236A | D | P | Ib | G | Gn | 3.8 | IV |
| 327 | PI 458248 | N | P | Y | G | Y | 4.3 | IV |
| 328 | PI 458253 | N | P | Br | T | Y | 4.0 | V |
| 329 | PI 458269 | D | P | Bf | G | Gn | 4.0 | IV |
| 330 | PI 458277 | D | P | Bf | G | Y | 3.0 | IV |
| 331 | PI 458288 | D | P | Br | T | Gn | 3.5 | IV |
| 332 | PI 458298 | D | W | Bl | T | Ggn | 4.3 | IV |
| 333 | PI 458301 | D | W | Bf | G | Y | 1.9 | IV |
| 334 | PI 458509 | D | W | Lbf | G | Y | 3.3 | IV |
| 335 | PI 458512 | D | P | Bf | G | Y | 1.8 | III |
| 336 | PI 458517 | N | W | Bf | G | Y | 5.0 | III |
| 337 | PI 458521 | N | P | Bl | T | Lgn | 5.0 | III |
| 338 | PI 464877 | N | P | Br | Ng | Br | 4.8 | III |
| 339 | PI 467312 | N | P | Br | T | Gnbr | 5.0 | II |
| 340 | PI 468384 | D | P | Y | G | Y | 2.5 | III |
| 341 | PI 468914 | D | P | Y | G | Y | 1.4 | III |
| 342 | PI 468915 | N | P | Bl | T | Bl | 3.3 | II |
| 343 | PI 468967 | N | P | Bl | Lt | Y | 4.0 | V |
| 344 | PI 470227B | D | P | Gn | G | Gn | 2.0 | III |
| 345 | PI 471931 | D | W | Y | G | Y | 2.5 | V |
| 346 | PI 476933 | S | P | Bf | G | Gn | 5.0 | V |
| 347 | PI 479740 | N | W | Y | G | Y | 3.3 | III |
| 348 | PI 483084 | D | P | Y | G | Y | 2.3 | IV |
| 349 | PI 495017C | N | P | Bl | T | Gn | 3.5 | IV |
| 350 | PI 504288 | N | Dp | Bl | T | Bl | 5.0 | V |
| 351 | PI 504509 | D | P | Br | T | Y | 2.0 | IV |
| 352 | PI 506420 | D | W | Tn | T | Y | 1.2 | IV |
| 353 | PI 506485 | D | W | Gn | G | Gn | 2.5 | V |
| 354 | PI 506524 | N | W | Tn | T | Y | 3.5 | IV |
| 355 | PI 506576 | D | P | Y | - | Y | 1.1 | IV |
| 356 | PI 506583 | D | W | Br | T | Y | 2.5 | V |
| 357 | PI 506592 | D | W | Bl | T | Bl | 1.1 | III |
| 358 | PI 506639 | N | W | Br | T | Gn | 5.0 | V |
| 359 | PI 506807 | N | P | Br | T | Y | 5.0 | V |
| 360 | PI 506924 | D | P | Brbl | T | Y | 2.5 | V |
| 361 | PI 506954 | D | P | Bl | T | Bl | 2.4 | IV |
| 362 | PI 506994 | D | W | Bl | T | Bl | 1.5 | IV |
| 363 | PI 507022 | N | W | Y | G | Y | 2.7 | IV |
| 364 | PI 507025 | D | P | Br | Lt | Gn | 1.6 | IV |
| 365 | PI 507060 | D | W | Br | T | Y | 3.6 | IV |
| 366 | PI 507066 | S | W | Br | T | Y | 2.9 | IV |
| 367 | PI 507098 | D | P | Y | G | Y | 3.0 | V |
| 368 | PI 507147 | D | W | Lbf | - | Lgn | 1.1 | III |
| 369 | PI 507151 | D | P | Y | G | Y | 3.0 | IV |
| 370 | PI 507152 | D | W | Y | G | Y | 2.5 | IV |
| 371 | PI 507153 | D | W | Lbf | G | Y | 2.3 | IV |
| 372 | PI 507158 | D | W | Y | G | Y | 2.5 | IV |
| 373 | PI 507226A | D | W | Bl | T | Bl | 1.4 | III |
| 374 | PI 507265 | D | P | Bf | G | Y | 2.6 | IV |
| 375 | PI 507267 | D | W | Bf | - | Y | 1.8 | III |
| 376 | PI 507283 | N | P | Br | T | Y | 5.0 | V |
| 377 | PI 507288 | D | W | Lbf | G | Y | 3.0 | V |
| 378 | PI 507307 | D | P | Y | G | Y | 3.5 | V |
| 379 | PI 507363 | D | P | Y | G | Y | 2.1 | IV |
| 380 | PI 507401 | D | P | Y | G | Y | 3.0 | V |
| 381 | PI 507411 | D | W | Y | G | Y | 3.2 | IV |
| 382 | PI 507416 | N | P | Y | G | Y | 4.5 | V |
| 383 | PI 507417 | N | P | Y | G | Y | 4.5 | V |
| 384 | PI 507420 | N | P | Y | G | Y | 4.5 | V |
| 385 | PI 507424 | N | W | Y | G | Y | 3.1 | IV |
| 386 | PI 507563 | D | P | Br | T | Y | 1.8 | IV |
| 387 | PI 507570 | D | W | Y | G | Y | 1.0 | III |
| 388 | PI 508296D | S | P | Bf | G | Y | 2.2 | IV |
| 389 | PI 509078 | D | P | Bl | T | Bl | 2.5 | V |
| 390 | PI 509112 | D | P | Bf | G | Gn | 3.2 | IV |
| 391 | PI 518757 | S | P | Br | T | Gn | 2.2 | III |
| 392 | PI 532462A | D | W | Lbf | G | Y | 1.2 | III |
| 393 | PI 538377 | D | P | Bf | G | Y | 1.5 | III |
| 394 | PI 548316 | N | W | Bl | Ng | Bl | 3.4 | III |
| 395 | PI 548317 | N | P | Bf | G | Gn | 3.8 | III |
| 396 | PI 548342 | D | P | Bf | G | Y | 2.0 | IV |
| 397 | PI 548349 | N | P | Br | Lt | Br | 3.8 | III |
| 398 | PI 548364 | N | W | Bf | G | Y | 3.0 | IV |
| 399 | PI 548402 | D | W | Bl | T | Bl | 2.7 | IV |
| 400 | PI 548415 | N | P | Bl | Ng | Bl | 4.0 | IV |
| 401 | PI 548464 | D | W | Gn | G | Gn | 1.0 | V |
| 402 | PI 549018 | N | W | Brbl | T | Gn | 5.0 | V |
| 403 | PI 549019 | N | W | Bl | T | Bl | 5.0 | V |
| 404 | PI 549026 | D | W | Bf | G | Y | 2.5 | V |
| 405 | PI 549031 | N | P | Bl | Lt | Bl | 4.0 | III |
| 406 | PI 549041A | N | P | Bl | T | Bl | 5.0 | III |
| 407 | PI 549045A | N | P | Bl | T | Bl | 5.0 | IV |
| 408 | PI 561271 | N | W | Bf | G | Gn | 5.0 | V |
| 409 | PI 561362 | D | P | Bf | G | Y | 2.0 | V |
| 410 | PI 561392 | D | P | Y | G | Y | 2.0 | V |
| 411 | PI 561395 | D | P | Y | G | Y | 2.5 | V |
| 412 | PI 561398 | D | W | Y | G | Y | 2.5 | V |
| 413 | PI 567183 | D | W | Br | T | Y | 4.0 | V |
| 414 | PI 567233 | N | P | Bf | G | Y | 5.0 | V |
| 415 | PI 567305 | N | P | Bl | T | Bl | 4.2 | IV |
| 416 | PI 567318 | D | W | Br | T | Gnbr | 4.2 | IV |
| 417 | PI 567336A | N | P | Bl | T | Bl | 4.5 | IV |
| 418 | PI 567343 | N | P | Br | Lt | Br | 3.5 | V |
| 419 | PI 567347 | D | P | Dbf | G | Gn | 1.5 | V |
| 420 | PI 567366A | N | P | Bl | T | Y | 3.5 | III |
| 421 | PI 567381B | D | W | Bf | G | Y | 2.0 | V |
| 422 | PI 567387 | N | P | Br | Lt | Gnbr | 4.5 | IV |
| 423 | PI 567395 | N | P | Bf | G | Ggn | 4.0 | IV |
| 424 | PI 567397 | D | W | Brbl | T | Y | 3.0 | V |
| 425 | PI 567482C | N | W | Lbf | G | Y | 3.8 | IV |
| 426 | PI 567488C | N | W | Dbf | G | Y | 2.5 | IV |
| 427 | PI 567507B | D | W | Bf | G | Y | 2.0 | II |
| 428 | PI 567516C | N | P | Gnbr | Lt | Gnbr | 4.8 | IV |
| 429 | PI 567521 | D | W | Bf | G | Y | 1.5 | V |
| 430 | PI 567561 | N | W | Bf | G | Y | 4.0 | IV |
| 431 | PI 567568B | N | W | Br | Lt | Y | 5.0 | V |
| 432 | PI 567583A | S | W | Bf | G | Y | 2.0 | III |
| 433 | PI 567595A | S | P | Bl | T | G | 3.5 | III |
| 434 | PI 567614B | D | W | Bf | G | Bf | 4.2 | IV |
| 435 | PI 567616 | S | P | Bf | G | Y | 4.0 | IV |
| 436 | PI 567619 | S | P | Bf | G | Y | 4.0 | III |
| 437 | PI 567627C | N | W | Bf | G | Y | 4.5 | IV |
| 438 | PI 567641 | S | W | Bf | G | Y | 4.5 | IV |
| 439 | PI 567671C | N | P | Br | Lt | Gnbr | 4.5 | IV |
| 440 | PI 567679B | N | P | Br | Lt | Y | 4.0 | IV |
| 441 | PI 567691 | S | P | Bf | G | Y | 4.5 | IV |
| 442 | PI 567693 | N | P | Bf | G | Y | 4.5 | IV |
| 443 | PI 567694 | N | W | Bf | G | Y | 4.8 | IV |
| 444 | PI 567696B | N | P | Bf | G | Y | 3.2 | IV |
| 445 | PI 567719 | N | W | Bf | G | Y | 4.5 | IV |
| 446 | PI 567721 | S | W | Bf | G | Y | 4.5 | IV |
| 447 | PI 567729 | S | Dp | Bf | G | Y | 4.5 | III |
| 448 | PI 567730 | S | W | Bf | G | Y | 4.0 | IV |
| 449 | PI 567734 | N | W | Lbf | G | Y | 4.8 | IV |
| 450 | PI 567741 | N | W | Br | T | Y | 3.0 | IV |
| 451 | PI 567744B | N | P | Bf | G | Y | 4.5 | IV |
| 452 | PI 578366 | S | W | Bf | G | Y | 3.0 | III |
| 453 | PI 578367 | D | W | Y | G | Y | 2.5 | III |
| 454 | PI 578439 | S | P | Bl | T | Y | 4.2 | III |
| 455 | PI 578440 | D | P | Dbf | G | Y | 3.0 | IV |
| 456 | PI 578451 | D | P | Bl | T | Bl | 4.0 | IV |
| 457 | PI 578486 | D | P | Y | G | Y | 1.5 | III |
| 458 | PI 587588A | N | P | Br | Lt | Y | 4.5 | IV |
| 459 | PI 587600A | D | P | Brbl | T | Y | 2.5 | IV |
| 460 | PI 587607A | D | W | Bf | G | Y | 2.0 | IV |
| 461 | PI 587620A | D | W | Bf | G | Y | 2.0 | IV |
| 462 | PI 587716B | D | W | Bf | G | Y | 3.0 | IV |
| 463 | PI 587719B | D | W | Brbl | T | Y | 3.0 | V |
| 464 | PI 587848 | S | P | Rbr | T | Rbr | 3.5 | V |
| 465 | PI 587860 | D | P | Bf | G | Y | 3.0 | V |
| 466 | PI 587975 | D | W | Bf | G | Y | 2.0 | V |
| 467 | PI 587979B | D | P | Ib | G | Y | 1.5 | IV |
| 468 | PI 587986B | S | W | Br | Lt | Y | 1.5 | V |
| 469 | PI 587998A | D | P | Bf | G | Y | 1.5 | IV |
| 470 | PI 588007A | D | W | Bf | G | Y | 3.0 | V |
| 471 | PI 588011A | D | W | Br | Lt | Y | 1.5 | V |
| 472 | PI 588015A | D | W | Brbl | T | Y | 2.0 | IV |
| 473 | PI 588016 | D | W | Bf | G | Y | 2.5 | IV |
| 474 | PI 588026C | D | W | Br | T | Y | 2.2 | IV |
| 475 | PI 588052B | D | P | Bl | T | Y | 2.0 | IV |
| 476 | PI 593988 | D | P | Bl | T | Gnbl | 1.0 | V |
| 477 | PI 594001 | D | P | Gn | T | Gn | 3.0 | V |
| 478 | PI 594006 | D | W | Bl | T | Gn | 2.0 | V |
| 479 | PI 594010 | D | P | Rbr | T | Rbr | 2.0 | IV |
| 480 | PI 594011 | D | W | Bl | T | Bl | 1.8 | IV |
| 481 | PI 594013 | S | P | Bl | T | Bl | 4.5 | V |
| 482 | PI 594022 | D | W | Br | T | Y | 1.5 | IV |
| 483 | PI 594023A | N | P | Bl | T | Bl | 2.5 | V |
| 484 | PI 594156 | D | P | Tn | - | Y | 1.0 | III |
| 485 | PI 594212 | D | P | Bl | T | Bl | 1.0 | V |
| 486 | PI 594267 | D | W | Y | G | Y | 1.0 | V |
| 487 | PI 594289 | D | P | Y | G | Y | 1.2 | IV |
| 488 | PI 594410 | D | P | Bf | G | Y | 1.5 | IV |
| 489 | PI 594471C | S | W | Brbl | T | Y | 2.5 | IV |
| 490 | PI 594480A | N | P | Bl | T | Gn | 3.2 | V |
| 491 | PI 594567D | D | P | Lbf | G | Y | 1.0 | V |
| 492 | PI 594568A | D | P | Lbf | G | Y | 1.0 | V |
| 493 | PI 594614B | D | W | Br | T | Y | 3.0 | V |
| 494 | PI 594621 | N | P | Bf | G | Gn | 4.0 | IV |
| 495 | PI 594650B | D | W | Br | T | Y | 3.0 | V |
| 496 | PI 594651 | D | W | Bf | G | Y | 2.5 | V |
| 497 | PI 594662B | D | W | Bf | G | Y | 3.0 | V |
| 498 | PI 594779 | D | W | Br | T | Y | 1.8 | IV |
| 499 | PI 594784B | D | P | Br | T | Y | 4.5 | V |
| 500 | PI 594856 | D | P | Rbr | T | Rbr | 1.0 | V |
| 501 | PI 594864 | D | P | Rbr | T | Rbr | 4.0 | V |
| 502 | PI 597474 | D | P | Y | G | Y | 2.2 | IV |
| 503 | PI 597481 | N | P | Bf | G | Y | 2.0 | IV |
| 504 | PI 597482 | D | W | Bl | T | Y | 1.0 | III |
| 505 | PI 597486 | D | P | Ib | G | Y | 2.0 | IV |
| 506 | PI 602991 | D | W | Bl | T | Bl | 2.0 | IV |
| 507 | PI 602992 | D | W | Bf | G | Y | 3.5 | IV |
| 508 | PI 603154 | D | W | Bf | G | Y | 1.5 | V |
| 509 | PI 603155 | N | W | Br | T | Gn | 2.2 | IV |
| 510 | PI 603156 | D | W | Bf | G | Gn | 2.2 | IV |
| 511 | PI 603158 | N | P | Rbr | Lt | Rbr | 3.2 | IV |
| 512 | PI 603162 | N | W | Bl | Lt | Bl | 3.8 | IV |
| 513 | PI 603168 | N | P | Bl | T | Bl | 2.8 | V |
| 514 | PI 603170 | D | P | Rbr | T | Rbr | 2.2 | IV |
| 515 | PI 603171 | N | P | Rbr | T | Br | 3.0 | IV |
| 516 | PI 603172 | N | P | Br | T | Gn | 3.2 | IV |
| 517 | PI 603174A | N | W | Bl | Lt | Bl | 2.5 | IV |
| 518 | PI 603175 | D | P | Rbr | T | Rbr | 1.8 | IV |
| 519 | PI 603176A | N | P | Bl | T | Bl | 3.8 | IV |
| 520 | PI 603397 | D | P | Bl | T | Y | 1.2 | IV |
| 521 | PI 603413 | N | P | Br | T | Br | 2.5 | IV |
| 522 | PI 603428D | S | P | Bl | T | Bl | 3.8 | III |
| 523 | PI 603445B | N | P | Bl | T | Bl | 4.5 | IV |
| 524 | PI 603452 | N | W | Bf | G | Y | 3.5 | III |
| 525 | PI 603465B | D | W | Bl | T | Y | 2.5 | IV |
| 526 | PI 603487B | D | P | Bf | G | Gn | 2.8 | IV |
| 527 | PI 603502C | N | P | Bl | Lt | Bl | 4.2 | IV |
| 528 | PI 603503 | N | P | Rbr | T | Rbr | 5.0 | IV |
| 529 | PI 603527A | N | P | Bl | Lt | Bl | 3.0 | IV |
| 530 | PI 603530C | D | P | Rbr | T | Rbr | 4.0 | V |
| 531 | PI 603551C | D | P | Bl | T | Bl | 3.2 | IV |
| 532 | PI 603573B | N | W | Bf | G | Y | 3.5 | V |
| 533 | PI 603578 | N | W | Bf | G | Y | 3.5 | V |
| 534 | PI 603600 | D | P | Br | T | Y | 3.0 | IV |
| 535 | PI 603637B | D | W | Brbl | T | Gn | 4.0 | IV |
| 536 | PI 603655 | D | P | Rbr | Lt | Br | 2.8 | III |
| 537 | PI 603673C | S | P | Brbl | T | Y | 4.8 | IV |
| 538 | PI 603674 | S | P | Ib | G | Y | 3.8 | III |
| 539 | PI 603677A | D | P | Gn | G | Gn | 2.0 | V |
| 540 | PI 603694B | D | W | Bf | G | Y | 2.5 | IV |
| 541 | PI 603714 | S | W | Bf | G | Y | 2.0 | V |
| 542 | PI 603720 | N | W | Tn | T | Y | 3.5 | V |
| 543 | PI 603912 | D | P | Y | G | Y | 1.8 | III |
| 544 | PI 603913D | S | W | Y | G | Y | 1.5 | IV |
| 545 | PI 603914 | N | P | Br | T | Gn | 2.5 | IV |
| 546 | PI 603915C | D | P | Br | T | Y | 1.2 | III |
| 547 | PI 605792D | D | P | Y | T | Y | 2.5 | IV |
| 548 | PI 605827B | S | W | Brbl | T | Y | 4.0 | V |
| 549 | PI 605865A | D | P | Bf | G | Y | 4.0 | V |
| 550 | PI 606412 | N | P | Br | T | Y | 4.5 | V |
| 551 | PI 612611 | N | P | Rbr | Lt | Br | 3.2 | III |
| 552 | PI 612612B | D | P | Y | G | Y | 1.5 | IV |
| 553 | PI 612614 | D | P | Bf | G | Y | 2.0 | IV |

| **Descriptor** | **Code** | **Definition** |
| --- | --- | --- |
| **Stem growth habit** | D | Determinate |
|  | N | Indeterminate |
|  | S | Semi-determinate |
| **Flower color** | B | Blue |
|  | Dp | Dark purple |
|  | P | Purple |
|  | W | White |
| **Hilum color** | Bf | Buff |
|  | Bl | Black |
|  | Blbr | Black hilum w/ brown outer ring |
|  | Br | Brown |
|  | Brbl | Brown w/variation from Lbr to Bl |
|  | Dbf | Dark buff |
|  | G | Gray |
|  | Gn | Green |
|  | Gnbr | Green-brown |
|  | Ib | Imperfect black |
|  | Lbf | Light buff |
|  | Lbl | Light black |
|  | Lbr | Light brown |
|  | Lgn | Light green |
|  | Rbf | Red buff |
|  | Rbr | Red brown |
|  | Tn | Tan |
|  | Y | Yellow |
| **Pubescence color** | - | If form=C or density=G |
|  | G | Gray |
|  | Lt | Light tawny |
|  | Ng | Near Gray |
|  | T | Tawny |
| **Seed coat color** | Bf | Buff |
|  | Bl | Black |
|  | Br | Brown |
|  | G | Gray |
|  | Ggn | Grayish green |
|  | Gn | Green |
|  | Gnbl | Greenish-black |
|  | Gnbr | Greenish-brown |
|  | Lgn | Light green |
|  | Rbf | Red buff |
|  | Rbr | Red brown |
|  | Y | Yellow |
